# SREBP2-activated CNPY3 Phase Separation Promotes Colorectal Cancer by Enhancing MDM2-mediated p53 Degradation

**DOI:** 10.7150/ijbs.125792

**Published:** 2026-05-15

**Authors:** Xue Li, Min-Yue Yin, Yan-Fei Wei, Jie Xing, Qian Zhang, Ye Zong, Shu-Tian Zhang, Si-An Xie

**Affiliations:** 1 Department of Gastroenterology, Beijing Friendship Hospital, Capital Medical University, State Key Laboratory of Digestive Health, National Clinical Research Center for Digestive Diseases, Beijing Key Laboratory for Precancerous Lesion of Digestive Diseases, Beijing, 100050, China.; 2 Department of Gastroenterology and Hepatology, Sichuan Provincial People's Hospital, School of Medicine, University of Electronic Science and Technology of China, Chengdu, 610072, China.

**Keywords:** Colorectal Cancer, SREBP2, CNPY3, Cholesterol Metabolism, Liquid-liquid phase seperation, MDM2

## Abstract

Dysregulated cholesterol metabolism is a recognized metabolic hallmark of cancer. While the transcription factor SREBP2 is a master regulator of this pathway, how its activation converts metabolic stress into the development of carcinogenic signals in colorectal cancer (CRC) remains unclear. Through clinical and preclinical analyses, we first confirmed that hypercholesterolemia and elevated tumoral SREBP2 are hallmarks of CRC. Using multi-omics integration, we identified CNPY3 as a direct transcriptional target of SREBP2. Functionally, CNPY3 drives CRC cell proliferation, invasion, and tumor growth via a cholesterol synthesis-independent oncogenic program. Clinically, high CNPY3 expression robustly correlated with advanced disease and poor patient survival. Mechanistically, we discovered that CNPY3 undergoes liquid-liquid phase separation (LLPS), a property dependent on its intrinsically disordered C-terminal region. This LLPS capacity is essential for its oncogenic function, as it enables CNPY3 to enhance MDM2 phosphorylation at the activating Ser166 site and promote its nuclear translocation. Consequently, CNPY3 potentiates MDM2-mediated ubiquitination and degradation of the tumor suppressor p53. Genetic ablation of p53 completely abolished the pro-tumorigenic effects of CNPY3, confirming p53 as the critical downstream effector. Crucially, this axis specifically targets wild-type p53, having no effect on common p53 mutants. Pharmacological disruption of the MDM2-p53 interaction with Nutlin-3 effectively reversed CNPY3-driven malignancy both *in vitro* and *in vivo*. Our work unveils a SREBP2-CNPY3-MDM2-p53 signaling axis that links cholesterol metabolic dysregulation to p53 pathway inactivation in CRC. We further established that the oncogenic activity of CNPY3 is mediated through its biophysical property of LLPS. These findings nominate CNPY3 as a novel prognostic biomarker and a compelling therapeutic target for p53-wild-type CRC.

## Introduction

Colorectal cancer (CRC) is the second leading cause of global cancer mortality, with a concerning rise in early-onset cases underscoring the need for deeper biological insights 1,2. Despite advancements in surgery, chemotherapy, targeted and immunotherapy, advanced CRC still faces the challenges of limited efficacy and drug resistance, highlighting the urgency to deeply elucidate its pathological mechanisms and develop new strategies. A core hallmark of cancer is metabolic reprogramming, and dysregulated cholesterol homeostasis has gained prominence 3. Epidemiological and clinical evidence substantiates that elevated serum cholesterol is associated with an increased risk of CRC, and cholesterol-lowering agents like statins correlate with reduced incidence and improved patient outcomes 4,5. These observations strongly implicate intrinsic cholesterol metabolic rewiring as a pivotal event in colorectal tumorigenesis.

The sterol regulatory element-binding protein 2 (SREBP2) is the master transcriptional regulator of cholesterol biosynthesis. SREBP2 is frequently upregulated in cancers, including CRC, where its established oncogenic role has been almost exclusively attributed to its canonical function in generating lipids for membrane biogenesis and signaling platforms (6-8). Although SREBP2 is upregulated in CRC and supports tumor growth by fulfilling lipid demands, it remains unclear whether it also drives progression through cholesterol-independent mechanisms. Here, we identify the endoplasmic reticulum-associated protein canopy FGF signaling regulator 3 (CNPY3) as a pivotal SREBP2 target that bridges this gap. While CNPY3 has roles in immunity and has been implicated in various cancers, its function in CRC and its potential to link metabolic dysregulation to oncogenic signaling are entirely unknown 9.

A powerful mechanism for organizing intracellular biochemistry is liquid-liquid phase separation (LLPS), a physicochemical process through which biomolecules condense into membrane-less compartments to regulate diverse cellular functions 10. Proteins with intrinsically disordered regions (IDRs) often drive LLPS, and aberrant phase separation is increasingly linked to oncogenesis, influencing processes from transcription to post-translational modification 11,12. We discovered that CNPY3 harbors a C-terminal IDR and undergoes LLPS—a property essential for its oncogenic function. Mechanistically, CNPY3 condensates promote MDM2 phosphorylation at Ser166 and its nuclear translocation, leading to enhanced ubiquitination and degradation of wild-type p53. This work delineates a novel SREBP2-CNPY3-MDM2-p53 axis, revealing how cholesterol metabolic stress is transduced into p53 pathway inactivation via LLPS. Our findings expand the oncogenic paradigm of SREBP2 beyond lipid synthesis and nominate CNPY3 as a promising therapeutic target for p53-wild-type CRC.

## Materials and Methods

### Cell Culture and Transfection

HCT116 and RKO were purchased from the Cell Bank of Type Culture Collection of Chinese Academy of Sciences, Shanghai Institute of Biochemistry and Cell Biology. HCT116 cells were cultivated in McCoy's 5A (Eallbio, China). RKO cells were cultivated in high-glucose DMEM (Eallbio, China). These media formulations were augmented with 10% fetal bovine serum (OPCEL, China). Cells are placed in a 37 °C incubator with 5% CO_2_.

oeSREBP2-HA plasmid, CNPY3-3xFlag plasmid, MDM2-HA plasmid, p53-3xFlag plasmid, p53-HA plasmid, CNPY3-Del plasmid, HA-Ubi plasmid, CNPY3 promoter-luciferase plasmid, mutated CNPY3 promoter-luciferase plasmid, and the control vector plasmids were procured from Youbio (Changsha, China). Small interfering RNA targeting CNPY3 (PRAT4A siRNA (h)) were purchased from Santa Cruz (sc-95269). The sequences of siRNA were as followed: siSREBP2#1: sense: GCUGCAAUUUGUCAGUAAU, anti-sense: AUUACUGACAAAUUGCAGC. siSREBP2#2: sense: CGCAGACGAGGAUCAUCCA, anti-sense: GGAUGAUCCUCGUCUGCG. Transfection was carried out with sitran siRNA (TT320002, Origen) or Megatran 2.0 (TT210003, Origene).

### Western Blotting

Cells were lysed in RIPA Lysis Buffer (P0013C, Beyotime Biotechnology) supplemented with protease inhibitors. Protein concentrations were measured with the BCA reagent (BL521A, Biosharp). Equal amounts of the denatured proteins were electrophoresed on sodium dodecyl sulfate-polyacrylamide gel, transferred to a 0.22 μM polyvinylidene fluoride membrane (Bio-Rad, 1620177), blocked in 5% skim milk for 2 h, and incubated with primary antibody overnight at 4 ℃ and the corresponding HRP antibodies at room temperature for 2 h after washing primary antibody. The antibodies used are listed in Table S1.

### GST-Pulldown

Plasmids expressing the corresponding proteins were introduced into competent *E. coli* for culture. 0.5 mM IPTG was added to the culture medium and induced at 16 °C 180rpm for 16 hours. Bacterial lysates were purified using GST Mag-beads (Sangon, C65003-0010). Add the GST-magnetic beads to incubate GST-CNPY3 protein or GST protein with purified His-p53-SUMO protein (Shanghai Nearshore Co., Ltd. purified) or purified His-MDM2 protein (Sino biological Co., Ltd. M45-31BH) for 30 minutes at room temperature. Protein elution was performed after washing the beads, and the eluted supernatant was used for western blot detection.

### Real-Time Quantitative Polymerase Chain Reaction (qRT-PCR)

Total RNA was extracted using Trizol (15596018, Thermo Fisher Scientific). Reverse-transcribe 0.5 μg RNA into cDNA using the PrimeScript Reagent Kit with gDNA Eraser (PR036A, Takara). Relative expression of mRNAs was assessed using the 2^-ΔΔCt^ method and standardized to GAPDH. Primers designed were listed in Table S2.

### Chromatin Immunoprecipitation (ChIP)-PCR

ChIP assays were conducted using kits from Beyotime. HCT116 cells (1 x 10^7^) were crosslinked with 1% formaldehyde, lysed, and genomic DNA was sheared by sonication. Anti-SREBP2 antibodies were used to precipitate DNA fragments. Quantitative real-time PCR was performed to determine the fold-enrichment of each fragment, with input DNA serving as a control. Primer sequences are listed in Table S2.

### 5-Ethynyl-2′-Deoxyuridine (EdU) Staining

Cells were cultured overnight on 12-well plates, with 1×10^5^ cells/well, and incubated with 50 μM EdU-A solution at 37 °C for 2 h. Other procedures followed the manufacturer's protocol of Cell-Light EdU Apollo567 *In vitro* Kit (C10310, RIOBOBIO).

### Colony Formation Assay

Transfected cells were seeded into 6-well plates at a density of 1000 cells/well, and cultured until a single colony formation larger than 50 cells. After fixation with 4% histiocyte fixative solution, the cells were incubated with 0.5% crystal violet for 0.5 h, and plates were washed in water to remove excess crystal violet.

### Wound Healing Assay

Culture fully confluent cells with 1% FBS in 6-well plates. Scrape out the equidistant wound inside the well using a sterile 200 μl pipette tip and photograph the same site at 0 h and 24 h, respectively.

### Transwell Migration Assay

Transwell chambers with 8-μM polycarbonate membrane (353097, Corning Costar) were placed in a 24-well plate. A total of 4×10^4^ cells were seeded with 200 μl of serum-free medium in the top chamber and 750 μl of complete medium was placed in the bottom chamber. After 48-hour incubation, the cells migrating into the lower surface were fixed with 4% histiocyte fixative solution and stained with 0.5% crystal violet in anhydrous methanol for 30 min.

### Apoptosis Assay

Flow cytometry was done to examine the apoptotic cells. Prepare samples according to the manufacturer's protocol of the FITC Annexin V Apoptosis Detection Kit (C1062L, Beyotime).

### Immunohistochemistry (IHC)

Paraffin-embedded slides were deparaffinized, rehydrated, transferred to Tris-EDTA buffer pH 9.0 or citrate buffer pH 6.0 for 3 min for antigen retrieval, and cooled in the antigen retrieval solution for 40 min. Goat serum (ZLI-9022, ZSGB-BIO) was used to block at room temperature for 20 min. Incubate slides with primary antibodies at 4 °C overnight, and the primary antibodies used were listed in Table S1. Then, the secondary antibody (PV-6000, ZSGB-BIO) was incubated at room temperature for 1 h. Then incubate slides with DAB (ZLI-9018, ZSGB-BIO) for 2.5 min at room temperature. The slides were counterstained with hematoxylin, dehydrated, and coverslip mounted.

### Immunofluorescence Staining (IF)

Samples were fixed with 4% histiocyte fixative solution and incubated with 0.3% Triton X-100 for 15 min at room temperature. 1% bovine serum albumin was used to block at room temperature for 20 min. Incubate samples with primary antibody at 4 °C overnight and fluorochrome-labeled secondary antibody for 2 h at room temperature the next day. DAPI (Santa Cruz Biotechnology, sc-24941) was used to identify cell nuclei. The primary antibodies are listed in Table S1.

### Co-Immunoprecipitation (Co-IP) Assay

Cells were lysed in Western and IP lysate buffer (P0013, Beyotime) with protease inhibitor. 2 μg primary antibody was added to the lysis buffer and incubated at 4 °C overnight. Protein A+G Magnetic Beads slurry (P2108, Beyotime) was used to capture the immunocomplex for 2 h at room temperature. Alternatively, cell lysis was added into anti-Flag Nanobody Magarose Beads (KTSM 1338, AlpaLifeBio) for 3 h at 4 °C under gentle agitation. Then wash beads with 1 ml 0.5% PBST for 5 min and repeat 4 times. Add 50 μl 1X SDS loading buffer and heat at 95 °C for 5 min to elute the pellet. The supernatant was taken for western blot detection. The antibodies of Co-IP are listed in Table S1.

### Animal Tumor Model

Animal experiments in this study were approved by the Animal Experiments and Experimental Animal Welfare Committee of Capital Medical University (approval number: AEEI-2023-286) and xenograft tumor diameters did not exceed 15 mm under the ethical guidelines. Six-week-old female BALB/c Nude mice were purchased from Beijing Vital River Laboratory Animal Technologies Co. Ltd. They were injected with 2*10^6^ HCT116 cells in 100 μl phosphate buffer saline (PBS). Tumor volumes were measured every 2-3 days, and the tumor volume was estimated according to the formula length diameter/2 * short diameter * short diameter. The AOM/DSS model was based on a single injection with AOM (10mg/kg, i.p., Aladdin) and a single cycle of 2% DSS (MeilunBio, China) in drinking water. Eight weeks after the injection, the mice were randomly assigned into Saline and Fatostatin (15 mg/kg) groups. The mice were sacrificed, and necropsies were performed after six-week treatment.

### Bioinformatics Analysis

Pan-cancer analysis of CNPY3 using the cBioPortal database (https://www.cbioportal.org/. The transcriptome data of the CPTAC colorectal cancer dataset were used for differential expression analysis of CNPY3. GEO database analysis: Four datasets were selected from the GEO database (www.ncbi.nlm.nih.gov/geo/): GSE6988, GSE20842, GSE23878, and GSE113513, and the original CEL file was downloaded from the GEO database, and differentially expressed genes were analyzed through the GEO2R platform (www.ncbi.nlm.nih.gov/geo/geo2r). The expression levels of CNPY3 in tumor tissues and normal tissues in COAD and READ datasets were analyzed online on the GEPIA website (gepia2.cancer-pku.cn). SREBP2 survival analysis and CNPY3 survival analysis associated with P53 stratification were from Kaplan-Meier Plotter 13. TCGA database analysis: Download the gene expression and clinical data of COAD and READ and analyze the differential expression of CRC-related genes using the "limma" software package in R language. GSEA analysis: TCGA differentially expressed genes were imported into the GESA analysis website (www.omicshare.com/tools/Home/Soft/gsea).

### Retrospective Cohort Study of CRC patients

This study retrospectively analyzed our CRC cohorts captaining 124 CRC patients, 81 healthy control and 163 adenoma patients, who tested blood lipid and triglyceride before colonoscopy examination. Human study was approved by the ethics committee at the Beijing Friendship Hospital, Capital Medical University [2020-P2-290-01] and conducted under the principles of the Declaration of Helsinki. Informed consent was waived by the Institutional Ethics Committee of Beijing Friendship University, Capital Medical University because of the retrospective nature of this study.

### Cell Titer Glo (CTG) assay

Cell viability was accessed by CTG assay according to the manufacturer's protocol. Briefly, HCT116 and RKO cells were seeded in 96 well white culture plates at a density of 1000 cells/well in 200 μl of the cell culture medium. Cells were incubated at 37 °C, 5% CO_2_ for 24 hours. Following incubation, 100 μl of media was added to each well and treated with 100 μl of CTG reagent and kept on a shaker for 2 minutes. The plates were kept at room temperature for 10 minutes to stabilize the luminescence signal. Luminescence was measured using microtiter plate ELISA reader (Bio Tek, Winooski, Vermont, USA).

### Phase Separation Assay

The full-length CNPY3 cDNA was cloned into the pET28a plasmid to express GFP-tagged fusion proteins. To assess the phase separation capability of CNPY3, the purified proteins were diluted in phase separation buffer (12.5 mmol/L HEPES, pH 7.5, and 0.5 mmol/L dithiothreitol) to the indicated concentrations. Subsequently, the diluted proteins were mixed with buffers containing 10% PEG8000, 5% 1,6-hexanediol, NaCl (ranging from 37.5 to 500 mmol/L), or water, and the formation of biomolecular condensates was observed in real time using a laser scanning confocal microscope (TCS SP8, Leica).

### Fluorescence Recovery After Photobleaching (FRAP) Assay

Cells were cultured in confocal dishes and transfected with GFP-CNPY3 plasmids for 48 hours. Imaging was performed using a laser scanning confocal microscope (STELLARIS, Leica). For FRAP analysis, selected condensates were photobleached with a 488 nm laser at 50% intensity for 3 seconds. Fluorescence recovery was monitored by acquiring images at 2-second intervals for 60 seconds post-bleaching. Fluorescence intensities within the bleached region were normalized to pre-bleach values to quantify the recovery kinetics.

### Statistical Analysis

Data for at least three independent experiments were expressed as mean ± standard deviation (SD). Center values were defined as mean and error bars in the scatterplots represent SD. Independent samples *t*-test, paired sample *t*-test and Wilcoxon Signed-Rank test were used to analyze the two groups. One-way and two-way analysis of variance (ANOVA) with post-hoc test was used to compare multiple groups. GraphPad Prism 9 was used for statistical analysis and visualization. The statistically significant difference was showed two-tailed *P < 0.05. Image information was collected using ImageJ.

## Results

### SREBP2-Mediated Cholesterol Synthesis Promotes Colorectal Tumor Growth

**Figure 1:**
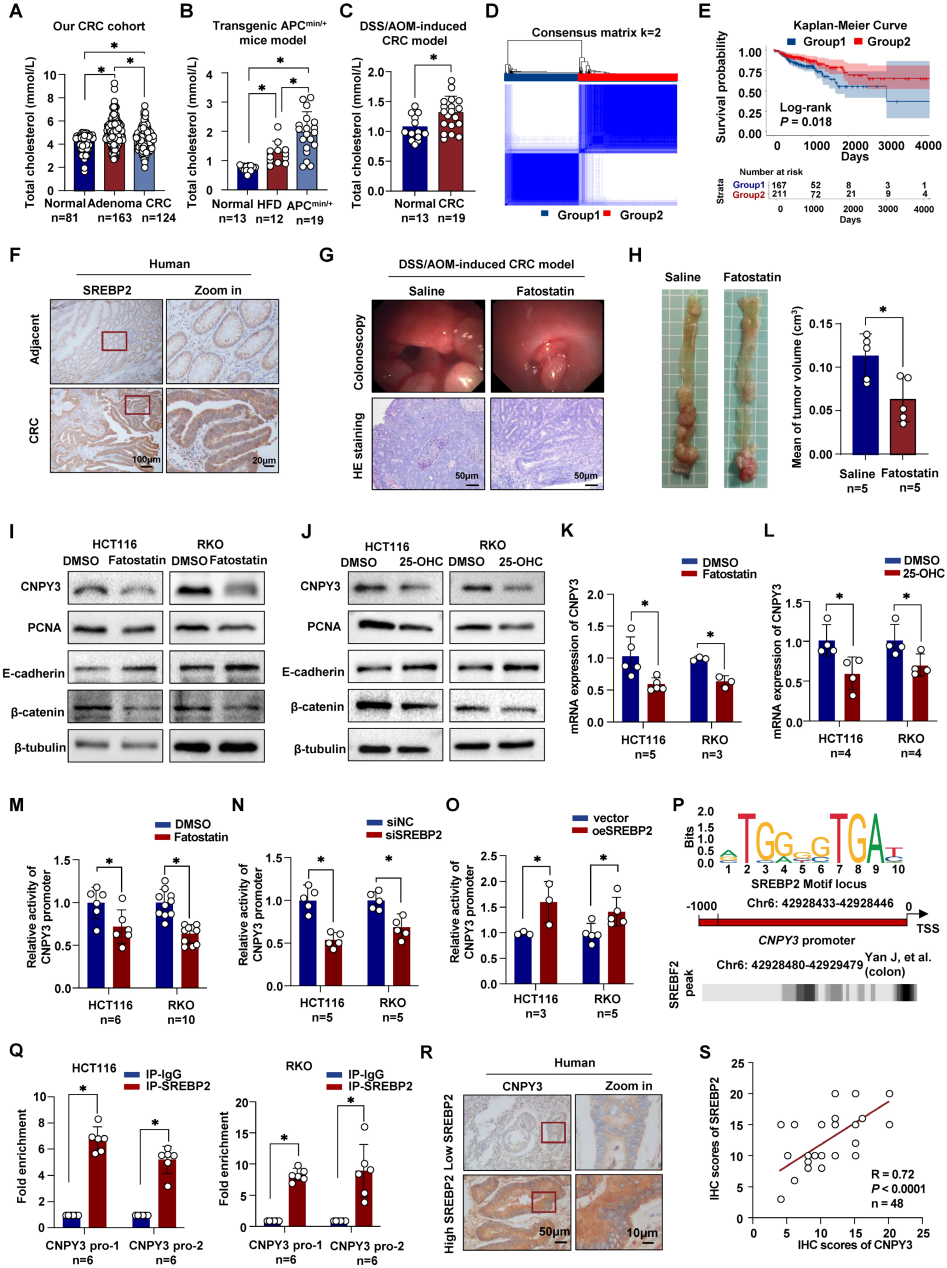
** Dysregulation of cholesterol homeostasis mediated by SREBP2 promotes colorectal tumor growth. (A-C)** Histograms show the levels of serum total cholesterol in our CRC cohort (A), transgenic APC^min/+^ mice model (B), and DSS/AOM-induced CRC mice (C). **(D)** Unsupervised consensus clustering defines two molecular subtypes based on cholesterol synthesis gene expression in CRC from the TCGA-COAD dataset. **(E)** Kaplan-Meier survival curve of overall survival analysis of two subtypes using log-rank test. **(F)** Representative IHC images of SREBP2 in CRC tissues and paired adjacent normal tissues from our CRC cohort. **(G-H)** The representative colonoscopy images and HE images (G), and mean tumor volume (H) of the DSS/AOM-induced CRC mice treated with Saline or Fatostatin (Selleck, S9785; 15mg/kg). **(I-J)** Western blot of CNPY3, PCNA, E-cadherin and β-catenin in HCT116 and RKO with Fatostatin treatment (MedChemExpress, HY-14452; 10 μM, 24h) (I) and 25-OHC (MedChemExpress, HY-113134; 10 μM, 24h) **(J)**, using β-tubulin as a control. **(K-L)** Histograms of mRNA expression of CNPY3 in HCT116 and RKO with Fatostatin treatment (K) and 25-OHC (L), using GAPDH as a control. **(M-O)** Luciferase reporter assays in HCT116 and RKO cells show that CNPY3 promoter activity is inhibited by the SREBP2 inhibitor Fatostatin (M) or by siSREBP2 knockdown (N) but is enhanced upon SREBP2 overexpression (O). **(P)** Schematic of the predicted SREBP2 binding motif within the human CNPY3 promoter. **(Q)** ChIP-qPCR assays using an anti-SREBP2 antibody confirm the enrichment of SREBP2 at the CNPY3 promoter region in HCT116 and RKO cells, compared to control IgG. **(R)** Representative IHC of CNPY3 in CRC tissues from patients with low and high SREBP2 levels. **(S)** Spearman's correlation analysis of SREBP2 and CNPY3 in CRC patients. Note: two-tailed *P < 0.05 by a Tukey's multiple comparisons test (A, B) or an unpaired *t* test (C, H, K, L, M, N, O, Q); 25-OHC, 25-Hydroxycholesterol; data are presented as mean ± SD.

We performed a retrospective analysis of a cohort comprising 124 CRC patients, 163 adenoma patients, and 81 healthy controls. Analysis of serum total cholesterol revealed significantly elevated levels in both adenoma and CRC groups compared to healthy controls (Figure 1A). Consistent with this clinical observation, elevated serum total cholesterol (TC) levels were also observed in genetically engineered APC^min/+^ mice (Figure 1B) and chemically DSS/AOM-induced CRC mice model (Figure 1C). To investigate the role of *de novo* cholesterol synthesis in CRC progression, we performed unsupervised consensus clustering based on the expression of key cholesterol synthesis genes in the TCGA-COAD dataset. This analysis stratified 483 tumors into two distinct subtypes: a cholesterol synthesis-high group (Group 1, 43.1%, n = 208) and a cholesterol synthesis-low group (Group 2, 56.9%, n = 275) (Figure 1D and Table S3). Patients in Group 1 exhibited significantly worse overall survival compared to those in Group 2 (Figure 1E).

Given the central transcriptional role of SREBP2 in cholesterol synthesis, we examined its protein expression in our patient cohort via IHC. SREBP2 expression is higher in tumor tissues than in adjacent normal tissues, and CRC patients with high SREBP2 expression have a worse prognosis (Figure 1F and Figure S1A). To functionally validate the oncogenic role of SREBP2 *in vivo*, we treated DSS/AOM-induced CRC mice with Fatostatin, a specific SREBP2 inhibitor. Colonoscopy prior to endpoint analysis revealed reduced luminal bleeding in Fatostatin-treated mice. Tumors in control mice appeared fragile with irregular surfaces and bled upon contact, whereas Fatostatin treatment resulted in fewer and less hemorrhagic lesions (Figure 1G). Histopathological quantification confirmed a significantly lower tumor burden in the Fatostatin-treated group (Figure S1B). Consistently, Fatostatin treatment led to a significant reduction in both tumor volume and serum TC levels (Figure 1H and Figure S1C). Collectively, these findings identify systemic hypercholesterolemia and tumor-intrinsic SREBP2 activation as key features of CRC and demonstrate that pharmacologic inhibition of SREBP2 suppresses tumor growth in preclinical models.

### SREBP2 Transcriptionally Activates CNPY3 to Drive CRC Progression

To elucidate the molecular mechanism of SREBP2-mediated conversion of cholesterol anabolic stress into pro-tumor signaling, we sought to identify its direct transcriptional targets. Cross-referencing differentially expressed genes in CRC (from TCGA and GEO databases) with genes that are both SREBP2-regulated and localized to the endoplasmic reticulum (ER) revealed CNPY3 as a top candidate (Figure S2A and Table S4). While CNPY3 has been implicated in colon cancer, its role in CRC remains unknown. We first examined whether CNPY3 expression is responsive to cholesterol synthesis inhibition. Treatment of CRC cells with inhibitors Fatostatin or 25-Hydroxycholesterol (25-OHC) significantly downregulated the expression of proliferation markers (PCNA, β-catenin) and increased expression of the epithelial marker E-cadherin (Figure 1I-J). This downregulation was accompanied by reduced expression of CNPY3 levels (Figure 1I-L and Figure S2B). Reciprocally, overexpression of SREBP2 upregulated CNPY3 (Figure S2C-D), while SREBP2 knockdown reduced its expression (Figure S2E-F). Luciferase reporter assays using the human CNPY3 promoter confirmed its transcriptional activation by SREBP2 (Figure 1M-O). Utilizing the JASPAR database and Cistrome Data Browser's data indicated that SREBF2 is a potential transcriptional regulator of CNPY3 (Figure 1P). Mutation of a putative SREBP2 binding site in CNPY3 promoter region predicted resulted in loss of the promoting (Figure S3). ChIP assays demonstrated direct binding of SREBP2 to the CNPY3 promoter region (Figure 1Q). In line with this regulatory relationship, IHC analysis of patient tissues showed that high SREBP2 expression correlated with significantly elevated CNPY3 protein levels (Figure 1R and Figure S4A). In our CRC cohort, there was a strong positive correlation between SREBP2 and CNPY3 expression (Figure 1S, R = 0.72, *P* < 0.0001) and between total cholesterol and CNPY3 expression (Figure S4B, R = 0.46, *P* = 0.0010). To determine if CNPY3 itself regulates cholesterol homeostasis, we manipulated its expression in CRC cells (Figure S5A-D) and assessed intracellular lipid droplet accumulation and total cholesterol levels. Neither CNPY3 knockdown nor overexpression significantly altered these parameters (Figure S5E-F), indicating that the function of CNPY3 is not to regulate cholesterol biosynthesis. Together, these data establish CNPY3 as a key transcriptional target of SREBP2 that mediates its oncogenic effects.

### CNPY3 is Overexpressed in CRC and Predicts Poor Prognosis in CRC patients

**Figure 2:**
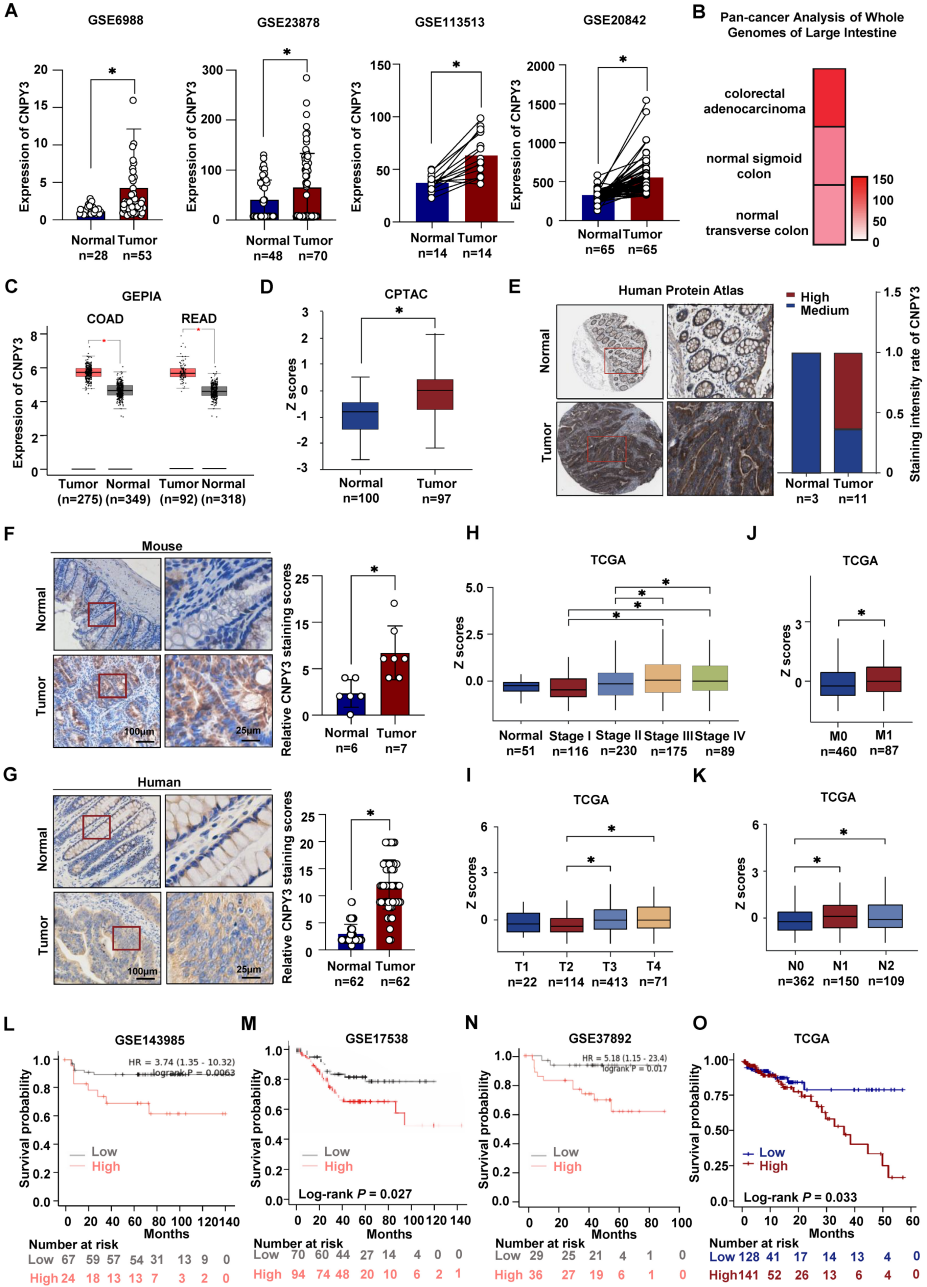
** CNPY3 is highly expressed in tumor tissues and worsens the prognosis of CRC patients. (A)** Relative CNPY3 expression levels were analyzed in CRC tissues and adjacent normal tissues from the GSE6988, GSE23878, GSE113513 and GSE20842 datasets. **(B)** Heatmap of CNPY3 mRNA level in Pan-cancer analysis of whole genomes of large intestine. **(C)** Boxplot of CNPY3 mRNA expression of COAD and READ from the GEPIA database. **(D)** Boxplot of CNPY3 protein levels from the CPTAC database. **(E)** Representative imagines of CNPY3 protein levels in CRC tissues and normal tissues from Human Protein Atlas (left) and semi-quantitative statistics (right). **(F)** Representative IHC images (left) and IHC analysis (right) of CNPY3 in CRC tissues and adjacent tissues of CRC mice. **(G)** Representative IHC (left) and IHC (left) analysis of CNPY3 in human CRC tissues and corresponding adjacent normal tissues using CRC tissue chips. **(H-K)** Boxplots of CNPY3 mRNA expression from TCGA database in tumor stage (H), T stage (I), M stage (J), and N stage (K). **(L-O)** Kaplan-Meier survival curve of overall survival analysis of CRC patients stratified by the cut-off value of CNPY3 mean using log-rank test from GSE143985 (L), GSE17538 (M), GSE37892 (N) and TCGA (O), respectively. Note: two-tailed *P < 0.05 by an unpaired *t* test (A, C, D, F, J, G) or a Wilcoxon Signed-Rank test (H, I, J, K); COAD, colon adenocarcinoma; READ, rectum adenocarcinoma; data are presented as mean ± SD.

We comprehensively analyzed CNPY3 expression across multiple independent cohorts. Meta-analysis of four GEO datasets (GSE6988, GSE20842, GSE23878, GSE113513) consistently demonstrated significant upregulation of CNPY3 mRNA in CRC tissues compared to normal controls (Figure 2A) 14-17. The upregulation of CNPY3 was also observed in CRC in the Pan-Cancer Analysis of Whole Genomes (Figure 2B) and the GEPIA (Figure 2C). At the protein level, the CPTAC database revealed aberrant elevation of CNPY3 in CRC (Figure 2D), and strong positive staining was observed in CRC specimens from the Human Protein Atlas (Figure 2E). Immunohistochemical validation confirmed that CNPY3 was remarkably elevated in CRC tissues compared to adjacent normal tissues in both CRC mice (Figure 2F) and CRC patients (Figure 2G). In pan-cancer analysis, CNPY3 was elevated in a variety of cancers (Figure S6A). Consistent upregulation was also observed in a panel of CRC cell lines versus normal colonic epithelial cells (Figure S6B-C). Clinically, CNPY3 expression exhibited a progressive increase with advancing TNM stage (Figure 2H). The levels of CNPY3 were significantly higher in advanced T stage (T3/T4 vs. T2, Figure 2I), M stage (M1 vs. M0, Figure 2J) and N stage (N1/N2 vs. N0, Figure 2K). Most importantly, survival analyses across multiple platforms consistently demonstrated that CRC patients with high CNPY3 expression had significantly shorter overall survival (Figure 2L-O). These multi-faceted data establish CNPY3 as a frequently overexpressed oncoprotein associated with aggressive disease and poor prognosis in CRC.

### CNPY3 Drives the Proliferation and Invasion of CRC

**Figure 3:**
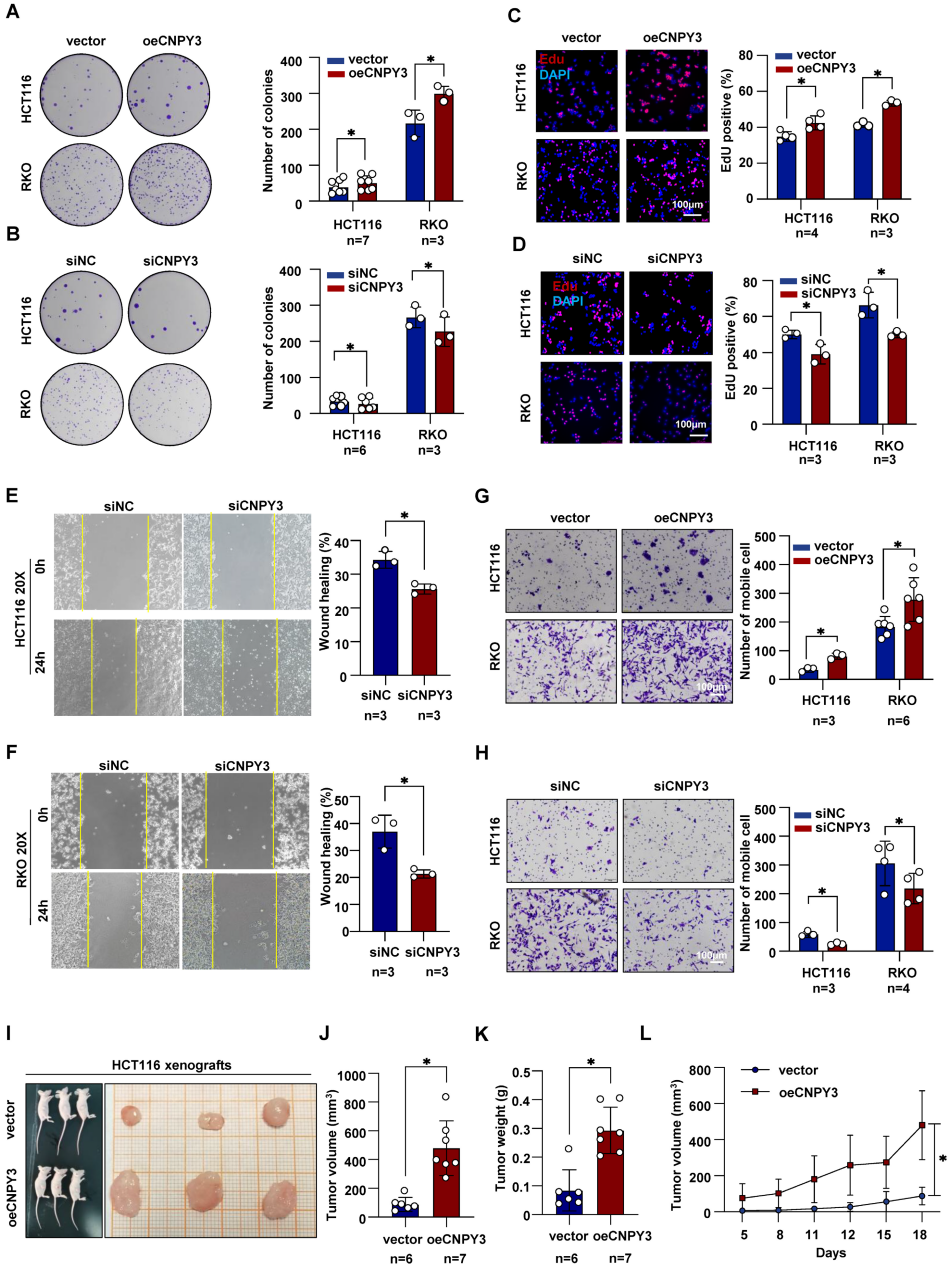
** CNPY3 facilitates the proliferation and invasion of CRC. (A-B)** Representative images (left) and quantitative statistics (right) of colony formation assay of HCT116 and RKO cells with CNPY3 overexpression (A) and CNPY3 knockdown (B). **(C-D)** Representative images of EdU incorporation (left) and quantification (right) of EdU-positive cells to DAPI-positive cells of HCT116 and RKO cells with CNPY3 overexpression (C) and CNPY3 knockdown (D). **(E-F)** Representative imagines of wound healing assay of HCT116 (E) and RKO (F) cells treated with CNPY3 knockdown (left) and quantitative statistics (right). **(G-H)** Representative images of migration assays (left) and quantification (right) of HCT116 and RKO cells with CNPY3 overexpression (G) and CNPY3 knockdown (H). **(I)** Representative images of subcutaneous xenograft tumors from BALB/c nude mice injected with vector-HCT116 and oeCNPY3-HCT116 cells. **(J-K)** Histogram of xenograft tumor volume (J) and tumor weight (K). **(L)** Tumor growth curves of tumor volumes assessed every 3 days. Note: two-tailed *P < 0.05 by an unpaired *t* test (J, K) and a Mix-effects analysis **(M)**; data are presented as mean ± SD.

We next performed functional assays to define the oncogenic role of CNPY3. Colony formation and EdU incorporation assays demonstrated that CNPY3 overexpression significantly enhanced the proliferative capacity of CRC cells, while CNPY3 knockdown markedly inhibited proliferation (Figure 3A-D). Similarly, wound healing and transwell invasion assays revealed that CNPY3 promoted cell migration and invasion, whereas its suppression had the opposite effect (Figure 3E-H and Figure S7). To assess its tumor-promoting function *in vivo*, we established xenograft models using CRC cells stably overexpressing CNPY3. Compared to control cells, CNPY3-overexpressing cells formed significantly larger tumors (Figure 3I-J), with increased tumor weight (Figure 3K), and accelerated growth kinetics over an 18-day period (Figure 3L). Additionally, functional rescue experiments demonstrated that the promoting effect of CNPY3-overexpressing on colony formation was reversed in cholesterol synthesis blockade CRC cells (Figure S8). In summary, CNPY3 is a potent driver of CRC malignant phenotypes.

### SREBP2-Induced CNPY3 Promotes Phosphorylation and Nuclear Translocation of MDM2

**Figure 4:**
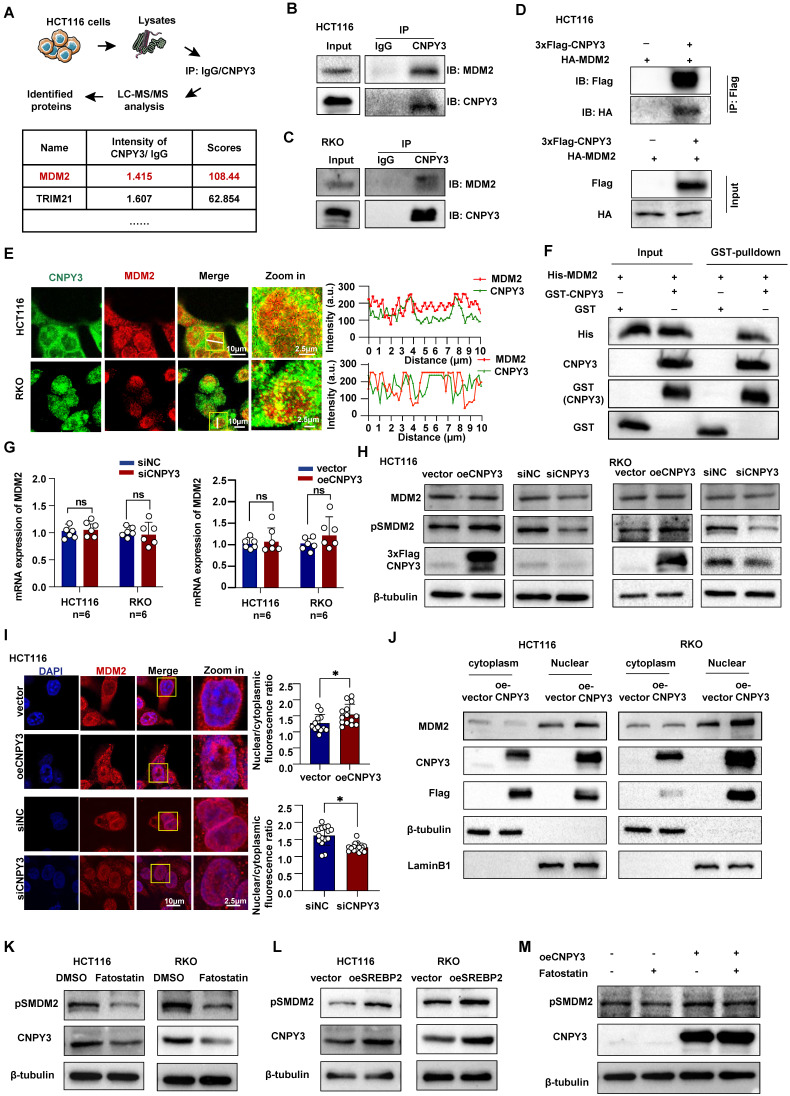
** SREBP2-CNPY3 axis promotes phosphorylation and nuclear translocation of MDM2. (A)** Flow chart of liquid chromatography-tandem mass spectrometry analysis and candidate proteins identified by CNPY3. **(B-C)** Endogenous Co-IP was performed in HCT116 (B) and RKO cells (C) lysates using an anti-CNPY3 antibody or control IgG. **(D)** Exogenous Co-IP analysis of Flag-CNPY3 and HA-MDM2 in HCT116 cells using anti-Flag nanobody agarose beads. **(E)** Representative IF staining images of colocalization between CNPY3 and MDM2 (left) and IF co-localization analysis (right) in HCT116 and RKO cells. **(F)** GST pull-down assay confirmed the direct interaction between purified his-tagged MDM2 protein and purified GST-tagged CNPY3 protein *in vitro*. **(G)** Histogram showed that MDM2 mRNA levels remain unchanged in HCT116 and RKO cells upon either knockdown (left) or overexpression (right) of CNPY3, compared to their respective controls. **(H)** Western blot showed MDM2, pSMDM2, and CNPY3 expression after CNPY3 overexpression or knockdown in HCT116 (left) and RKO cells (right), using β-tubulin as a control. **(I)** Representative IF staining (left) and semi-quantitative statistics (right) exhibited MDM2 sublocalization after overexpression of CNPY3 in HCT116 and RKO cells. **(J)** Cytoplasmic and nuclear protein isolation and western blotting were performed in HCT116 (left) and RKO cells (right) overexpressing CNPY3 or control. **(K-L)** Western blot showed pSMDM2 and MDM2 in HCT116 and RKO cells with Fatostatin treatment (K) and overexpression of SREBP2 (L), using β-tubulin as a control. **(M)** Western blot showed pSMDM2 and CNPY3 in CNPY3 overexpression HCT116 cells after Fatostatin treatment, using β-tubulin as a control. Note: two-tailed *P < 0.05 and ns by an unpaired *t* test (G, I); ns, not significant; pSMDM2, phosphorylated MDM2-Ser166; data are presented as mean ± SD.

To elucidate the mechanism through which CNPY3 drives tumor progression, we conducted KEGG pathway enrichment analysis on TCGA data stratified by CNPY3 expression. Pathways associated with endoplasmic reticulum protein processing and ubiquitin-mediated proteolysis were significantly enriched in the CNPY3-high group (Figure S9A). Using liquid chromatography-tandem mass spectrometry (LC-MS/MS), we identified the E3 ubiquitin ligase MDM2 as a major CNPY3-interacting protein (Figure 4A). This interaction was validated by Co-IP in CRC cells (Figures 4B-D). Confocal microscopy further demonstrated co-localization of CNPY3 and MDM2 in CRC cells (Figure 4E). A GST pull-down assay with purified proteins confirmed a direct physical interaction between CNPY3 and MDM2 (Figure 4F). We observed that CNPY3 did not affect MDM2 mRNA expression (Figure 4G) or total MDM2 protein levels (Figure 4H). Given that phosphorylation of MDM2 at Ser166 (pS166-MDM2) is essential for its nuclear translocation and E3 ligase activity, we next examined this post-translational modification. Modulation of CNPY3 expression specifically enhanced MDM2 phosphorylation at Ser166 without altering total MDM2 abundance (Figure 4H). Consistent with these findings, IF and cellular fractionation assays revealed that CNPY3 overexpression promoted nuclear accumulation of MDM2 (Figures 4I-J). To link this regulation to the upstream transcription factor SREBP2, we inhibited SREBP2 with Fatostatin, which resulted in downregulation of pS166-MDM2 (Figure 4K). Conversely, SREBP2 overexpression increased pS166-MDM2 levels (Figure 4L). Notably, this effect was not observed with the SQLE inhibitor Terbinafine (Figure S9B). Importantly, concurrent overexpression of CNPY3 rescued the Fatostatin-induced suppression of MDM2 phosphorylation (Figure 4M). Collectively, these results define a signaling axis in which SREBP2 upregulates CNPY3, which subsequently interacts with MDM2 and promotes its activating phosphorylation and nuclear translocation.

### The Oncogenic Function of CNPY3 is Dependent on CNPY3 LLPS Capacity

**Figure 5:**
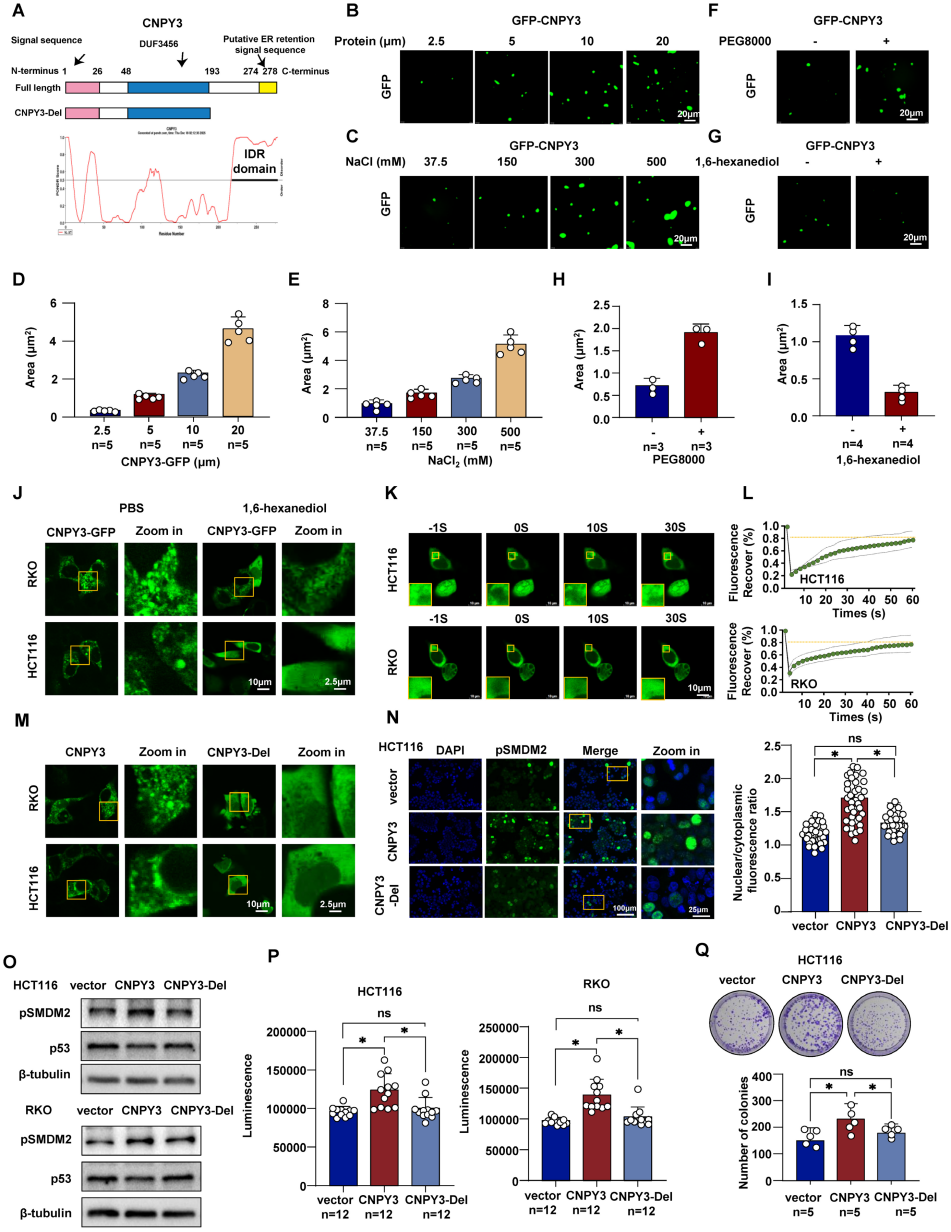
** LLPS of CNPY3 drives oncogenic function by promoting MDM2 phosphorylation. (A)** Schematic representation of the domain architecture of human CNPY3 and the deletion mutant (CNPY3-Del) used in this study. **(B-C)** Representative confocal microscopy images showed that purified GFP-tagged CNPY3 is concentration-dependent (B) and enhanced by higher salt concentrations (C). **(D-E)** Quantification of CNPY3 droplet formation demonstrates its dependence on both protein concentration (D) and salt concentrations (E). **(F)** Representative images showed that treatment with the crowding agent 10% PEG8000 (Biosharp, 0159) promotes the formation of liquid droplets by purified GFP-CNPY3. **(G)** Representative images demonstrated that treatment with 5% 1,6-hexanediol (MACKLIN, H810887) effectively disrupts GFP-CNPY3 liquid droplets. **(H-I)** Quantification of CNPY3 droplet formation after PEG8000 (H) and 1,6-hexanediol treatment (I). **(J)** Representative confocal images of HCT116 and RKO cells treated with 1,6-hexanediol. **(K-L)** Representative images (K) and recovery curves (L) from a fluorescence recovery after photobleaching (FRAP) experiment show rapid and substantial fluorescence recovery within the photobleached CNPY3-GFP droplet. **(M)** Representative confocal images of HCT116 and RKO cells transfected with CNPY3-GFP and CNPY3-Del plasmids. **(N)** Representative imagines (left) and semi-quantitative analysis (right) of pSMDM2 in HCT116 cells with overexpression CNPY3-GFP or CNPY3-Del plasmids, compared with control plasmids. **(O)** Western blot showed pSMDM2 and p53 expression after overexpression of CNPY3 or CNPY3-Del plasmids in HCT116 (left) and RKO cells (right), using β-tubulin as a control. **(P)** Histogram of CTG luminescence in HCT116 (left panel) and RKO cells (right panel) with vector, CNPY3 and CNPY3-Del plasmid overexpression. **(Q)** Colony assay and quantitative analysis of HCT116 cells with vector, CNPY3 and CNPY3-Del plasmid overexpression. Note: two-tailed *P < 0.05 and ns by a Tukey's multiple comparisons test (N, P, Q); ns, not significant; pSMDM2, phosphorylated MDM2-Ser166; data are presented as mean ± SD.

LLPS of oncoproteins is emerging as a crucial mechanism for their function. As the presence of IDRs is a hallmark of proteins capable of undergoing LLPS, we analyzed CNPY3 and identified a predicted disordered region at its C-terminus (Figure 5A). We therefore investigated whether CNPY3 possesses LLPS properties. Using purified GFP-tagged CNPY3 protein for *in vitro* reconstitution, we observed the formation of micrometer-sized liquid droplets via confocal microscopy. These droplets increased in both size and number with higher protein concentration or in solutions with higher salt concentration (Figure 5B-E). Additionally, treatment of PEG8000 enhanced the GFP-CNPY3 LLPS, whereas 1,6-hexanediol abolished the droplet formation of GFP-CNPY3 (Figure 5F-I). Furthermore, we detected CNPY3 status in CRC cells. Endogenous CNPY3 formed punctate condensates (Figure S10), and transfected CNPY3-GFP formed larger, dynamic condensates (Figure 5J). FRAP experiments confirmed the liquid-like nature of these condensates, as evidenced by rapid fluorescence recovery (Figure 5K-L).

To test the functional importance of LLPS, we generated a C-terminal IDR deletion mutant (CNPY3-Del). Live imaging results showed that CNPY3-del failed to form condensates and exhibited a diffuse distribution (Figure 5M). Importantly, this LLPS-deficient mutant lost the ability to promote MDM2 Ser166 phosphorylation (Figure 5N). CNPY3-del failed to upregulate MDM2 Ser166 phosphorylation or decreased p53 (Figure 5O). Functional assays showed that full-length CNPY3 overexpression accelerated cell proliferation in CTG and colony-formation assays, whereas CNPY3-Del lost this capacity (Figure 5P-Q). These findings demonstrate that CNPY3 undergoes functional LLPS, and this biophysical property is essential for its ability to activate MDM2 and drive oncogenic phenotypes.

### SREBP2-CNPY3 Axis Accelerates MDM2-Mediated Ubiquitination and Degradation of p53

As the principal E3 ligase responsible for ubiquitinating and degrading the tumor suppressor p53, MDM2 was further investigated in the context of CNPY3 regulation. GSEA of TCGA data revealed significant suppression of the p53 signaling pathway in CNPY3-high tumors (Figure S11A), which was corroborated by KEGG analysis of cholesterol synthesis subtypes (Figure S11B). We next performed structural modeling of the CNPY3-MDM2-p53 complex, which computationally supported the formation of a trimeric assembly (Figure S12A). Docking analysis identified multiple potential hydrogen-bonding interactions between CNPY3 and MDM2 (Figure S12A and Table S5). Co-IP and IF staining further confirmed co-localization of CNPY3 with p53 (Figure S12B-C).

**Figure 6:**
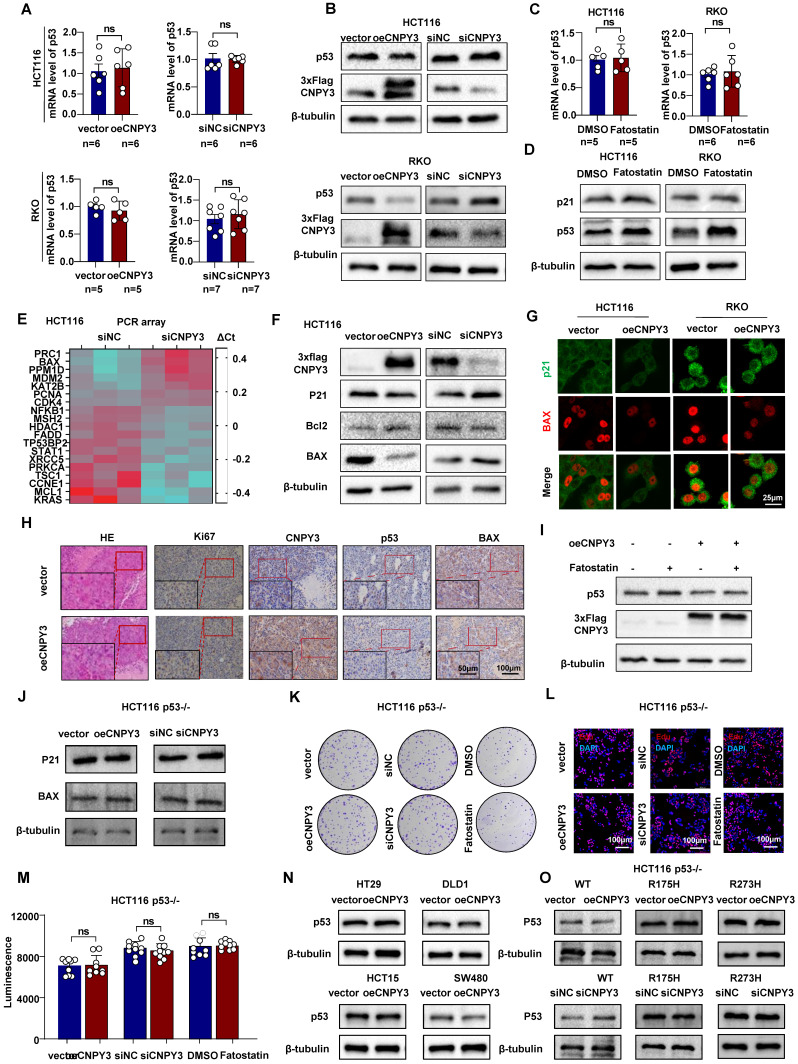
** CNPY3 reduced the protein level of wild-type p53. (A)** Histograms of p53 mRNA expression in response to manipulated CNPY3 in HCT116 (up) and RKO cells (down), using GAPDH as a control. **(B)** The p53 and CNPY3 protein expression after manipulating CNPY3 in HCT116 (up) and RKO cells (down), using β-tubulin as a control. **(C)** Histograms of p53 mRNA expression in HCT116 (left) and RKO cells (right) treated with Fatostatin, using GAPDH as a control. **(D)** Protein expression of p53 and p21 in HCT116 and RKO cells with Fatostatin treatment, using β-tubulin as a control. **(E)** Heatmap showed that CNPY3 regulated mRNA expression of genes related to p53 signaling pathway from PCR array analysis (WC-MRNA0117-H, Wcgene). **(F)** Western blot showed CNPY3, p21, Bcl2 and Bax in HCT116 cells with CNPY3 knockdown and CNPY3 overexpression, using β-tubulin as a control. **(G)** IF staining exhibited p21 (green) and BAX (red) after CNPY3 overexpression in HCT116 and RKO cells. **(H)** Representative images of IHC staining of HE, Ki67, CNPY3, p53, and BAX expression in HCT116 tumor xenografts. **(I)** Western blot showed p53 and CNPY3 in CNPY3 overexpression HCT116 cells after Fatostatin treatment, using β-tubulin as a control. **(J)** Western blot showed p21 and Bax in p53-/- HCT116 cells with CNPY3 knockdown or overexpression, using β-tubulin as a control. **(K)** Colony formation assay of p53-/- HCT116 cells treated with CNPY3 knockdown, CNPY3 overexpression, or Fatostatin treatment. **(L)** Representative images of EdU incorporation of p53-/- HCT116 cells treated with CNPY3 knockdown, CNPY3 overexpression, or Fatostatin treatment. **(M)** Histogram of CTG luminescence in p53-/- HCT116 cells with CNPY3 knockdown, CNPY3 overexpression, or Fatostatin treatment. **(N)** Western blot showed p53 in HT29, DLD1, HCT15, and SW480 cells overexpressed CNPY3, using β-tubulin as a control. **(O)** Western blot overexpression of wild-type p53, R175H p53, or R273H followed by knockdown or overexpression of CNPY3 p53 levels in p53-/- HCT116 cells with changes in p53 levels, using β-tubulin as a control. Note: ns by an unpaired *t* test (A, C, M); ns, not significant; data are presented as mean ± SD.

Both qPCR and western blot analyses indicated that CNPY3 and SREBP2 regulate p53 exclusively at the post-translational level, without affecting its transcription (Figure 6A-D and Figure S13A-B). CNPY3 overexpression also reduced nuclear accumulation of p53 (Figure S13C). Transcriptomic profiling demonstrated that CNPY3 suppresses p53 transcriptional activity, as evidenced by downregulation of its canonical targets p21 and BAX and concomitant upregulation of the anti-apoptotic protein Bcl2 (Figure 6E-G). Consistent with these molecular changes, CNPY3 promoted CRC cell proliferation and migration (Figure 3), whereas CNPY3 knockdown induced apoptosis and its overexpression conferred resistance to apoptosis (Figure S14). *In vivo*, xenografts stably overexpressing CNPY3 exhibited decreased p53 and BAX protein levels along with elevated Ki67 expression (Figure 6H and Figure S15). Pharmacological inhibition of SREBP2 with Fatostatin restored p53 expression, an effect that was attenuated by concurrent CNPY3 overexpression (Figure 6I), confirming that CNPY3 acts downstream of SREBP2 to suppress p53.

To establish p53 as the essential effector in this pathway, we utilized CRISPR/Cas9-generated p53-knockout HCT116 cells (Figure S16A-B). In p53-null cells, neither CNPY3 nor SREBP2 modulated the expression of p53 target genes (BAX, p21) (Figure 6J) or influenced cell proliferation (Figures 6K-M and Figure S17). We further examined the specificity of this regulation toward wild-type p53. Modulation of CNPY3 in CRC cell lines harboring common p53 mutants (R273H, R175H) did not alter mutant p53 protein stability (Figure 6N). Reconstitution experiments in p53-null cells confirmed that CNPY3 selectively promoted the degradation of wild-type p53, but not the R175H or R273H mutants (Figure 6O). Furthermore, we found that in CRC patients with wild-type P53, high expression of CNPY3 often indicates a poor prognosis, while in CRC patients with mutant P53, CNPY3 expression is not related to the prognosis of CRC (Figure S18A-C).

**Figure 7:**
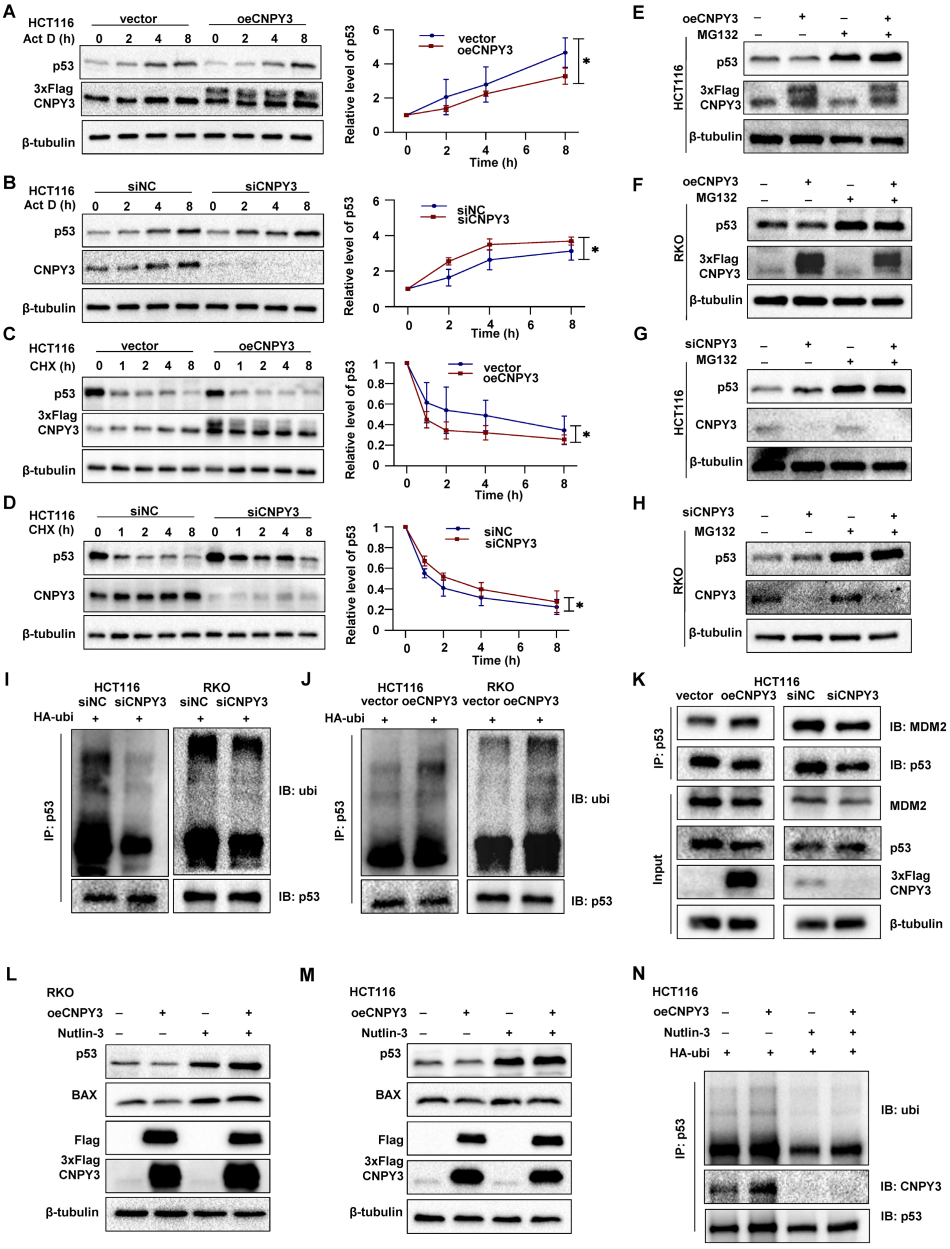
** CNPY3 promotes p53 ubiquitin-proteasome degradation. (A-B)** Act D-chase assays (Sigma, A4262; 5 μM) (left) and semi-quantitative statistical results (right) were used to evaluate CNPY3 accumulation in HCT116 cells with CNPY3 overexpression (A) and CNPY3 knockdown (B), using β-tubulin as a control. **(C-D)** CHX-chase assays (MedChemExpress, HY-12320; 100ug/ml) (left) and semi-quantitative statistical results (right) were used to evaluate CNPY3 degradation in HCT116 cells with CNPY3 overexpression (C) and CNPY3 knockdown (D), using β-tubulin as a control. **(E-F)** The p53 protein levels of CNPY3 overexpressing HCT116 (E) and RKO cells (F) with or without MG132 treatment (Selleck, S2619; 20 μM, 5 h), using β-tubulin as a control. **(G-H)** The p53 protein levels of CNPY3 knockdown HCT116 (G) and RKO cells (H) with or without MG132 treatment, using β-tubulin as a control. **(I-J)** Ubiquitination assays of endogenous p53 in the lysate from HA-ubi overexpressing HCT116 and RKO cells with CNPY3 knockdown (I) and CNPY3 overexpression (J). **(K)** Co-IP analysis of binding efficiency of p53 and MDM2 through p53-IP in HCT116 cells with CNPY3 knockdown and overexpression, compared with controls. **(L-M)** The protein levels of p53 and BAX in CNPY3 overexpression RKO (L) and HCT116 cells (M) after Nutlin-3 treatment, using β-tubulin as a control. **(N)** Ubiquitination assays of p53 in the lysate from HA-ubi overexpressing HCT116 cells with CNPY3 overexpression after Nultin-3 treatment. Note: two-tailed *P < 0.05 by a two-way ANOVA test (A, B, C, D); Act D, Actinomycin D; CHX, Cycloheximide; data are presented as mean ± SD.

Mechanistically, stable CNPY3 overexpression significantly accelerated p53 protein degradation under Actinomycin D or Cycloheximide treatment compared to controls, while CNPY3 knockdown had the opposite effect (Figure 7A-D). MG132-mediated proteasome blockade completely rescued CNPY3-dependent p53 degradation (Figure 7E-H). Ubiquitination assays confirmed that CNPY3 upregulated p53 ubiquitination levels (Figure 7I-J). Co-IP confirmed that CNPY3 enhanced the binding efficiency between MDM2 and p53 (Figure 7K). Furthermore, the MDM2-p53 interaction inhibitor Nutlin-3 not only restored p53 levels (Figure 7L-M) but also abolished CNPY3-augmented p53 ubiquitination (Figure 7N). Collectively, these results indicated that SREBP2 transcriptionally upregulates CNPY3, which via LLPS enhances MDM2 activity, leading to accelerated ubiquitination and degradation of wild-type p53, thereby promoting CRC progression.

### Therapeutic Targeting of MDM2 Attenuates CNPY3-Induced CRC Malignancy

**Figure 8:**
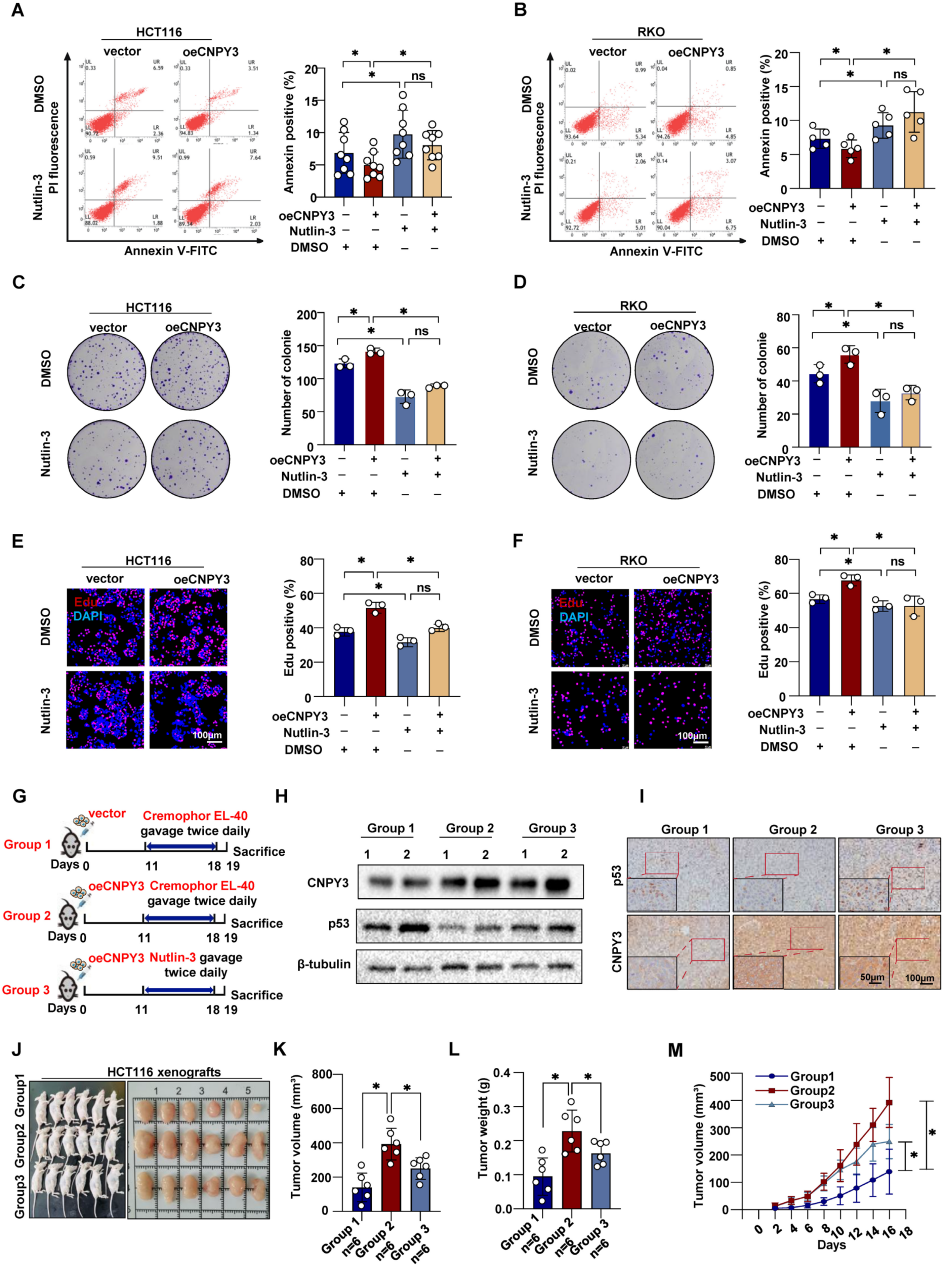
** Nutlin-3, a p53 degradation blocker, potently inhibits CNPY3 tumorigenesis. (A-B)** Apoptosis assays (left) and quantitative statistics (right) of HCT116 (A) and RKO cells (B) with vector or CNPY3 overexpression after treating DMSO or Nutlin-3. **(C-D)** Colony formation assay (left) and quantitative statistics (right) of HCT116 (C) and RKO cells (D) with vector or CNPY3 overexpression after treating DMSO or Nutlin-3. **(E-F)** EdU incorporation and quantification of EdU-positive cells to DAPI-positive cells of HCT116 (E) and RKO cells (F) with vector or CNPY3 overexpression after treating DMSO or Nutlin-3. **(G)** Schematic diagram shows that three xenograft tumor groups were treated with Cremophor EL-40 or Nutlin-3 (dissolved in Cremophor EL-40 and gavage twice daily at 150 mg/kg) after injecting HCT116 cells with vector or CNPY3 overexpression. **(H)** Western blotting of p53 and CNPY3 expression in three xenograft tumor groups, using β-tubulin as a control. **(I)** Representative imagines of IHC staining of CNPY3 and p53 in the three-group tissues. **(J)** Morphological images of xenograft tumors in the three groups **(K-L)** Histogram of tumor volume (K) and tumor weight (L) among the three groups. **(M)** Tumor growth curves of tumor volumes were assessed every 2 days, beginning at 4 days post-injection. Note: two-tailed *P < 0.05 and ns by a Tukey's multiple comparisons test (A, B, C, D, E, F, K, L) or a Mix-effects analysis (M); ns, not significant; data are presented as mean ± SD.

Given the malignancy characteristics associated with high CNPY3 expression, we next investigated whether pharmacological blockade of its downstream effector MDM2 could be therapeutically exploited in CNPY3-high CRC. *In vitro*, Nutlin-3 disrupted the MDM2-p53 axis and fully reversed CNPY3-mediated phenotypes. Blockade of MDM2-mediated p53 degradation rescued the inhibitory effect of CNPY3 on cell apoptosis (Figure 8A-B) and reversed the proliferative phenotype (Figure 8C-F). In orthotopic xenografts derived from CNPY3-overexpressing cells, continuous administration of Nutlin-3 markedly reduced tumor volume, tumor weight, and growth kinetics (Figure 8G-M). Collectively, these data demonstrate that MDM2 inhibition phenocopies CNPY3 loss and validates MDM2 as a tractable dependency in CNPY3-driven CRC. Treatment of CRC mice with Fatostatin resulted in significant inhibition of CNPY3 expression and elevation of p53 expression in the tumor tissues (Figure S19A-B). In addition, both Fatostatin and Nutlin-3 were found to significantly suppress the proliferation of colorectal cancer cells 18-19. The combined treatment of the two agents further enhanced this effect (Figure S19C-F). Targeting MDM2 with Nutlin-3 phenocopies CNPY3 loss to suppress CRC malignancy and combining Fatostatin with Nutlin-3 further enhances anti-proliferative and pro-apoptotic effects, supporting a synergistic therapeutic strategy for CNPY3-driven CRC.

## Discussion

Cholesterol dysregulation, a hallmark of malignancy, sustains tumor growth through enhanced membrane biogenesis and oncogenic signaling 20. The cancer-promoting effect of abnormal cholesterol metabolism in CRC has received great attention, and inhibition of cholesterol effectively promotes the anti-tumor immunity CRC 21,22. SREBP2 expression was shown to be significantly upregulated in colorectal cancer liver metastasis, and inhibition of SREBP2-dependent cholesterol biosynthesis pathway could effectively inhibit CRC liver metastasis 23. Although it is generally recognized that the imbalance of cholesterol homeostasis causes the abnormal elevation of SREBP2 to promote cancer, there is still a gap in the research on the regulation of tumor oncogenic factor activation/tumor suppressor factor inactivation by SREBP2 24,25. Our study bridges this translational gap by identifying CNPY3 as a non-canonical SREBP2 effector that couples' cholesterol dysregulation with p53 inactivation. CNPY3, as a downstream of SREBP2, effectively links the mevalonate pathway to ubiquitination, delineates the complex network of molecular mechanisms of tumor regulation, and enables a broader selection of therapeutic targets for CRC patients with lipid metabolism reprogramming.

CNPY3 is previously thought to be an ER chaperone, and few studies have focused on its role in tumors, except for two recent studies. One study showed that CNPY3 binding to SLITRK4 promotes liver metastasis of gastric cancer by promoting TrkB receptor endocytic recycling; the second study showed that gambogic acid recruits the deacetylase SIRT1, which abolishes lysine lactation on CNPY3, affecting cellular localization of CNPY3 to promote lysosomal rupture and induce prostate cancer pyroptosis 26,27. Although a recent study has shown that CNPY3 is highly expressed in colon cancer, which is closely related to tumor progression and poor prognosis, the specific molecular mechanism is unclear 28. Our study found for the first time that activated SREBP2 promotes transcriptional expression of CNPY3 due to cholesterol homeostasis dysregulation. Further exploration proves that CNPY3 is highly expressed in CRC and elevated CNPY3 is associated with poor prognosis in patients, while up-regulated CNPY3 promotes colorectal malignancy *in vivo* and *in vitro*. This is consistent with the results of the previously reported pro-cancer properties of CNPY3.

Emerging research provides evidence that LLPS has a critical role in human health and disease 29. Phase-separated multimolecular assemblies provide a universal regulatory mechanism for dividing intracellular biochemical reactions 30. Clarifying the experience of the LLPS protein that plays a decisive role in tumor growth could provide new avenues for potential therapeutic interventions. Our findings demonstrated that CNPY3 undergoes LLPS via its C-terminal IDR to promote MDM2 phosphorylation at Ser166, thereby providing a biophysical framework linking cholesterol metabolism to p53 inactivation. Previous study has shown that lipid deposition significantly enhances YTHDF3-mediated m6A modification and PPARα degradation, a process that reduces the β-hydroxybutyrylation of YTHDF3, thereby promoting LLPS and increasing the stability of YTHDF3, which in turn promotes the progression of CRC and liver metastasis 31. Our study demonstrated that cholesterol metabolism promotes CRC progression through LLPS of CNPY3, providing another paradigm for metabolic regulation of protein post-translational modification caused by LLPS. Post-translational modifications act as effective molecular switches that regulate various protein LLPS to participate in biological processes by affecting protein localization, conformation, physical interactions, abundance, or activity, and LLPS in turn can regulate protein post-translational modifications 32. This bidirectional complexity makes LLPS-based research more complex. We demonstrated that the LLPS of CNPY3, driven by its intrinsically disordered C-terminus, is indispensable for enhancing MDM2 phosphorylation at Ser166. The genetic ablation of the CNPY3 IDR, which abolished both phase separation and tumorigenic function, underscores the mechanistic necessity of LLPS. From a translational perspective, the dependence of this axis on CNPY3 condensation reveals a potential therapeutic vulnerability. Targeting the formation or output of these SREBP2/CNPY3-driven condensates could offer a novel strategy for treating p53-wild-type CRC, distinguishing it from broader LLPS inhibition approaches.

CNPY3 accelerated ubiquitinated proteasome degradation of p53 protein in an MDM2-dependent manner, leading to CRC progression. Nutlin-3 is a small-molecule *cis*-imidazoline compound and binds to the pocket of the p53 N-terminus to inhibit its interaction with the p53-MDM2 complex 33. RG7112 belonging to the Nutlin family could alleviate relapsed/refractory acute myeloid leukemia by activating p53, but it had serious gastrointestinal toxicity, including nausea, vomiting, and diarrhea 34. Thus, clinical trials of Nutlin-3 have not been satisfactory. Our data reveal its potent activity in CNPY3-high/p53-wild-type tumors, redefining patient stratification criteria. Especially for wild-type p53 patients with lipid metabolism, statins combined with p53 protein stabilizers are expected to dually block the progression of malignant tumors with high expression of CNPY3. Although there is still a long way to go from research to translation, it brings new therapeutic hope for clinical patients.

Although this study is a comprehensive and in-depth study of the molecular function of CNPY3, there are still certain limitations to the study. First, although we have demonstrated the pro-cancer effect of CNPY3 with overexpression, knockdown and overexpression rescue experiments, we lack CNPY3 knockout cell lines and CNPY3 knockdown rescue experiments to further validate. Second, due to the lack of a purified protein crystal structure of CNPY3, we were unable to achieve more precise protein sites and tertiary structure of CNPY3. Thirdly, although this project has in-depth proof of the mechanism of action of CNPY3 in promoting the development of CRC, there is a lack of drug development directly targeting CNPY3 for the treatment of CRC, and the future focusing on CNPY3 therapeutic targets is expected to provide new hope for CRC patients. Finally, due to the short follow-up time of our cohort and the temporary inability to conduct effective prognostic assessment, we hope to be able to supplement the follow-up data of colorectal cancer cohorts with CNPY3 as a prognostic marker in future studies. Nevertheless, this study demonstrates how cholesterol homeostasis could promote the development of CRC at the molecular level, and CNPY3 may become a new prognostic marker and provide a new and effective target for CRC treatment.

## Conclusions

**Figure 9:**
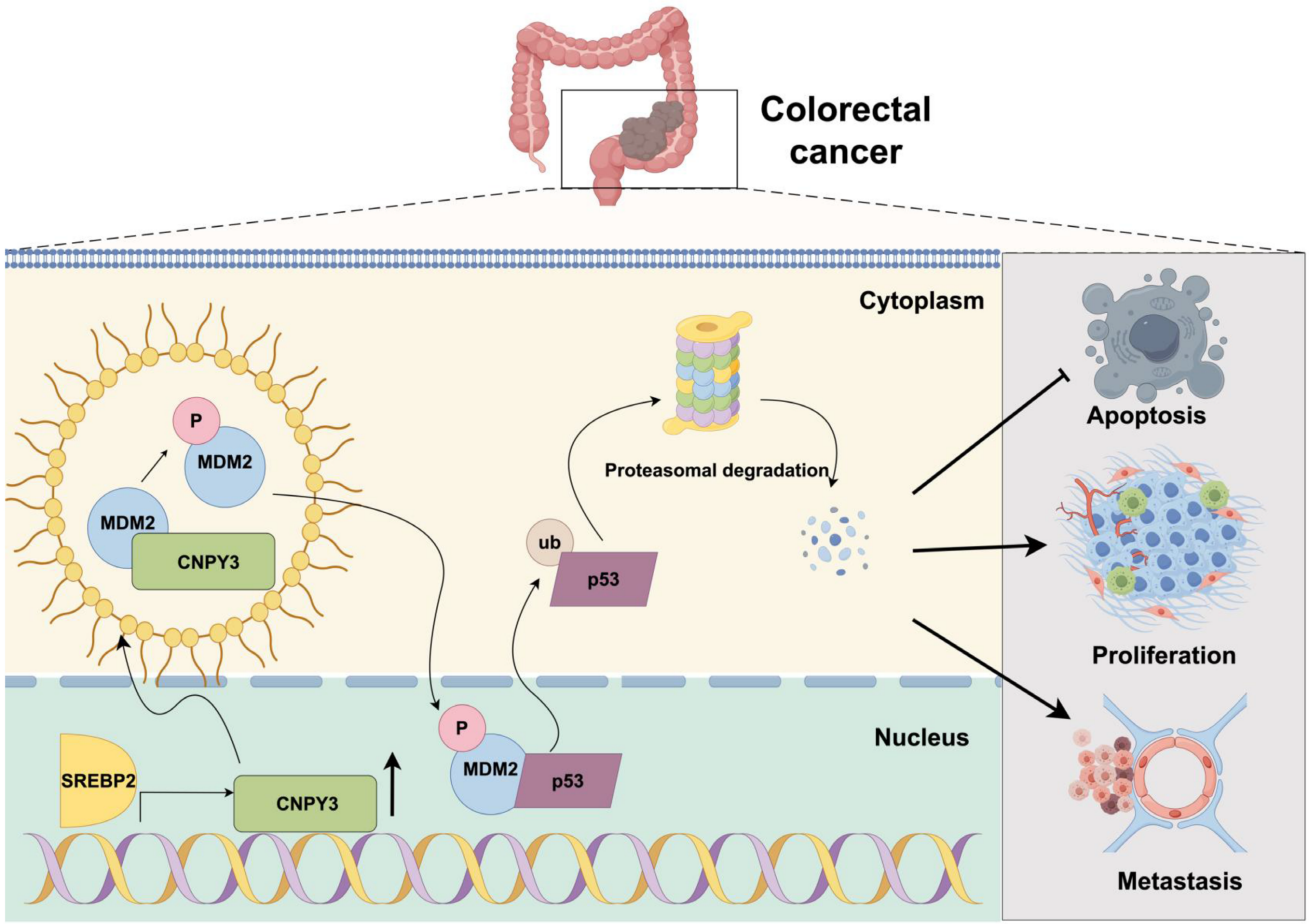
** Mechanism schematic diagram.** SREBP2-activated CNPY3 undergoes liquid-liquid phase separation to enhance MDM2 phosphorylation and nuclear translocation, thereby accelerating ubiquitination-mediated degradation of wild-type p53 and driving colorectal cancer progression.

In conclusion, our study delineates a coherent signaling pathway-from extracellular cholesterol dysregulation to intracellular tumor suppressor inactivation-that critically promotes CRC progression. We demonstrate that CNPY3, transcriptionally activated by SREBP2, undergoes LLPS via its C-terminal IDR to promote MDM2 phosphorylation at Ser166 that potentiates MDM2-mediated ubiquitination and degradation of wild-type p53, thereby enabling unchecked cellular proliferation and tumorigenesis (Figure 9). The dependence of this axis on wild-type p53 and its susceptibility to MDM2 inhibition with Nutlin-3 highlight its therapeutic relevance. This SREBP2-CNPY3-MDM2-p53 axis not only enriches our understanding of how metabolic reprogramming contributes to cancer pathogenesis but also provides a mechanistic rationale for targeting lipid metabolism in CRC treatment.

## Supplementary Material

Supplementary figures and tables.

## Data Availability

The datasets used and analyzed during the current study are available from the corresponding author on reasonable request.

## Abbreviations

CRC: colorectal cancer
LLPS: liquid-liquid phase separation
CNPY3: canopy FGF signaling regulator 3
SREBP2: sterol regulatory element-binding protein 2
IDR: intrinsically disordered regions
qRT-PCR: Real-time quantitative polymerase chain reaction
ChIP: Chromatin immunoprecipitation
EdU: 5-Ethynyl-2′-deoxyuridine
IHC: Immunohistochemistry
IF: immunofluorescence staining
Co-IP: Co-immunoprecipitation
CTG: Cell Titer Glo
FRAP: Fluorescence Recovery After Photobleaching
SD: standard deviation
ANOVA: analysis of variance
TC: total chelosterol
