# Supplementary material for: SREBP2-activated CNPY3 Phase Separation Promotes Colorectal Cancer by Enhancing MDM2-mediated p53 Degradation
Source: Int J Biol Sci. 2026 May 15;22(10):5301–19. doi: 10.7150/ijbs.125792 (PMC13215366; doi:10.7150/ijbs.125792)
Supplement: Supplementary file 1 — Supplementary figures and tables. [file ijbsv22p5301s1.pdf]

SREBP2-activated CNPY3 phase separation promotes colorectal cancer by enhancing MDM2-mediated p53 degradation

Supplementary Figures:

Figure S1 to S19

Supplementary Material 1 includes:

Table S1 to S5

Supplementary figure legends

Raw imagines

Figure S1

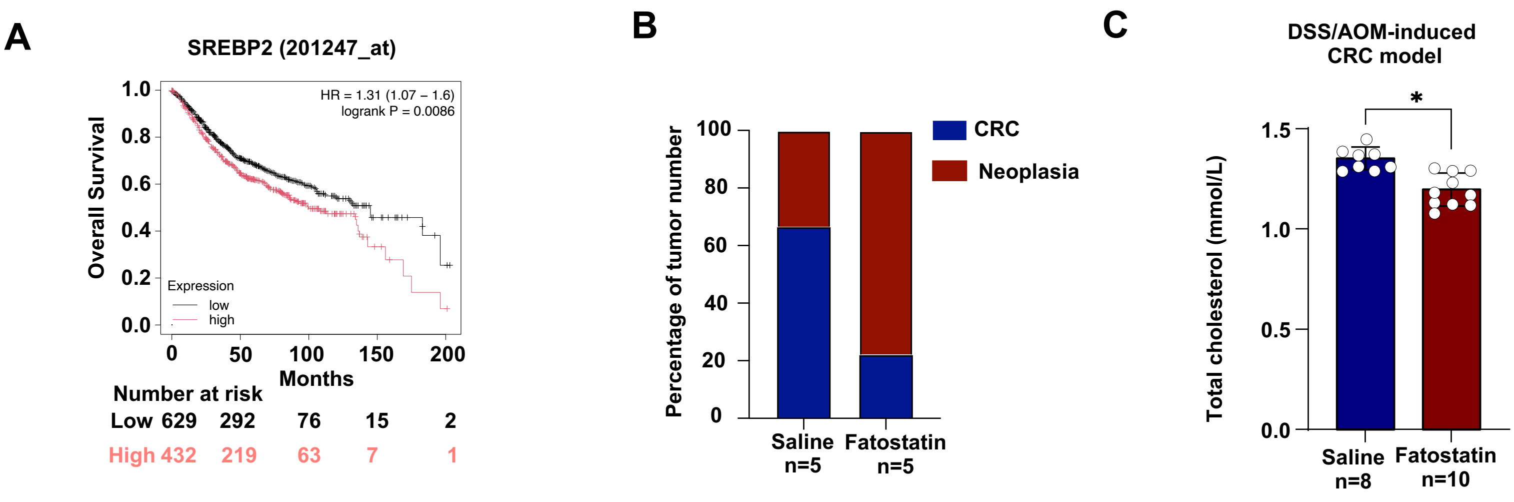
A
SREBP2 (201247_at)
HR = 1.31 (1.07 − 1.6)
logrank P = 0.0086
Overall Survival
1.0
0.8
0.6
0.4
0.2
0.0
Expression
low
high
0
50
100
150
200
Months
Number at risk
Low 629 292 76 15 2
High 432 219 63 7 1
B
Percentage of tumor number
100
80
60
40
20
0
CRC
Neoplasia
Saline n=5
Fatostatin n=5
C
DSS/AOM-induced CRC model
*
Total cholesterol (mmol/L)
1.5
1.0
0.5
0.0
Saline n=8
Fatostatin n=10

Figure S2

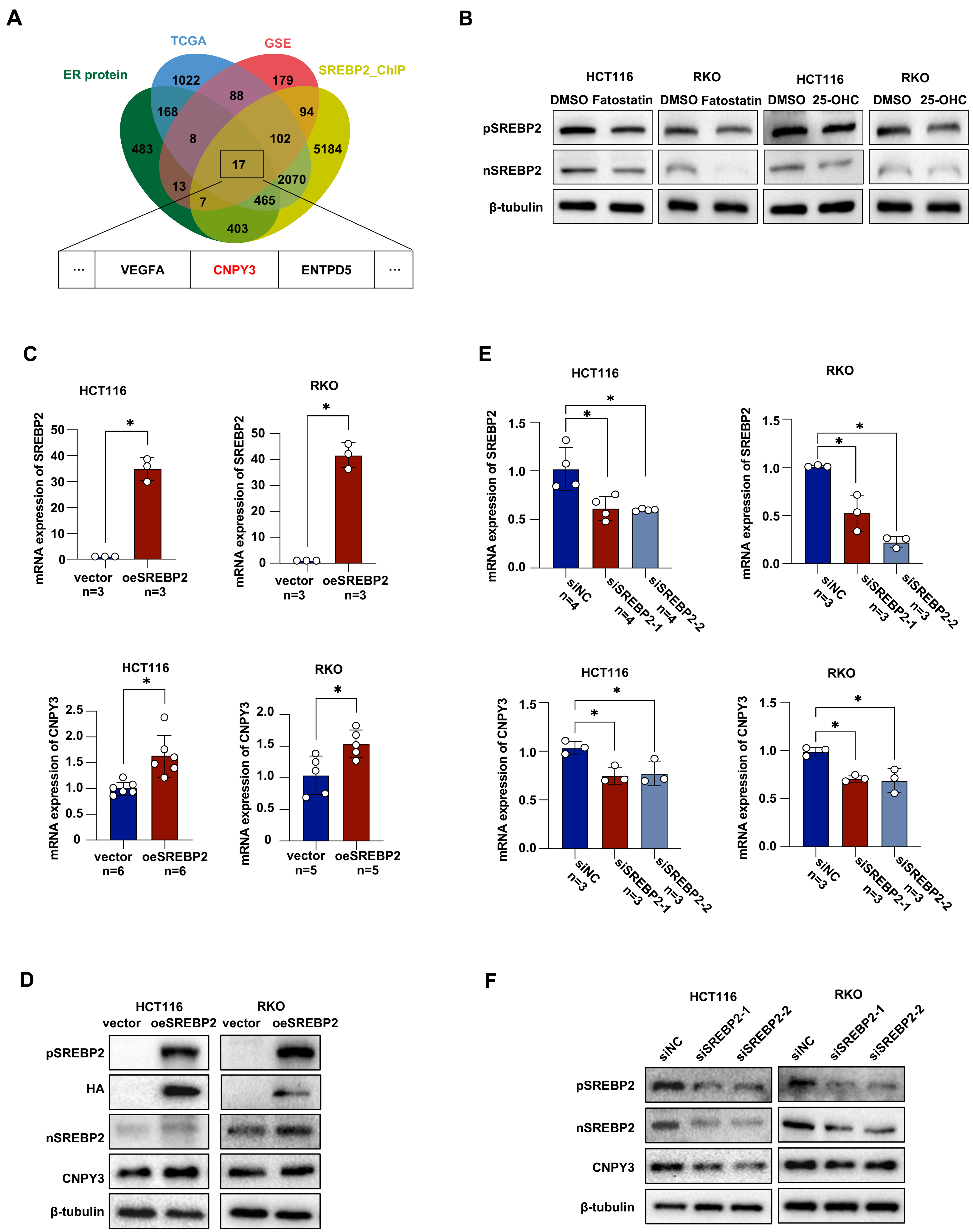
A
TCGA
GSE
ER protein
SREBP2_ChIP
1022
179
88
168
94
8
102
483
5184
17
13
2070
7
465
403
…
VEGFA
CNPY3
ENTPD5
…
B
HCT116
RKO
HCT116
RKO
DMSO Fatostatin DMSO Fatostatin DMSO 25-OHC DMSO 25-OHC
pSREBP2
nSREBP2
β-tubulin
C
HCT116
RKO
mRNA expression of SREBP2
vector n=3
oeSREBP2 n=3
HCT116
RKO
mRNA expression of CNPY3
vector n=6
oeSREBP2 n=6
vector n=5
oeSREBP2 n=5
E
HCT116
RKO
mRNA expression of SREBP2
siNC n=4
siSREBP2-1 n=4
siSREBP2-2 n=4
siNC n=3
siSREBP2-1 n=3
siSREBP2-2 n=3
mRNA expression of CNPY3
D
HCT116
RKO
vector oeSREBP2 vector oeSREBP2
pSREBP2
HA
nSREBP2
CNPY3
β-tubulin
F
HCT116
RKO
siNC
siSREBP2-1
siSREBP2-2
pSREBP2
nSREBP2
CNPY3
β-tubulin

Figure S3

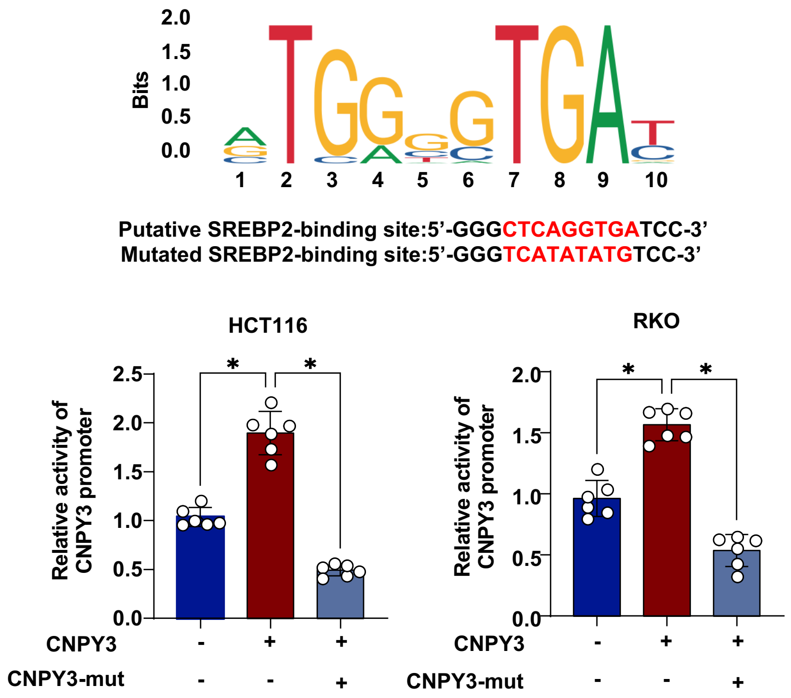

Bits
2.0
1.5
1.0
0.5
0.0
1 2 3 4 5 6 7 8 9 10
Putative SREBP2-binding site:5’-GGGCTCAGGTGATCC-3’
Mutated SREBP2-binding site:5’-GGGTCATATATGTCC-3’
HCT116
RKO
Relative activity of CNPY3 promoter
2.5
2.0
1.5
1.0
0.5
0.0
*
CNPY3 - + +
CNPY3-mut - - +

Figure S4

A

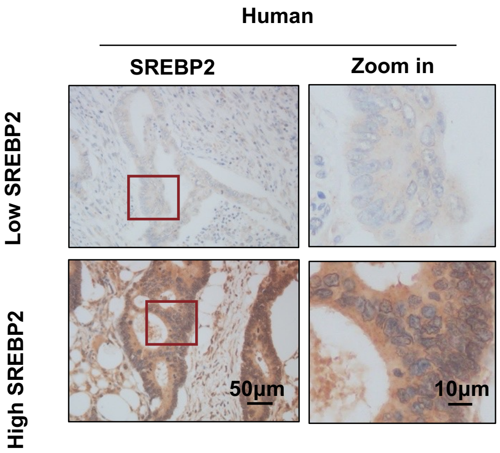
Human
SREBP2
Zoom in
Low SREBP2
High SREBP2
50μm
10μm

B

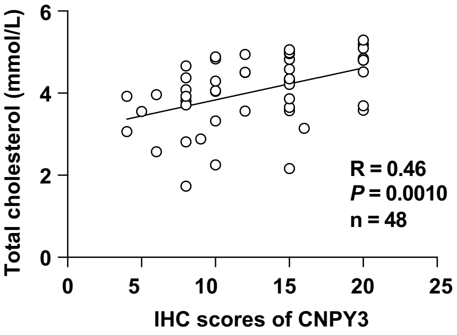
Total cholesterol (mmol/L)
IHC scores of CNPY3
R = 0.46
P = 0.0010
n = 48
0
2
4
6
0
5
10
15
20
25

Figure S5

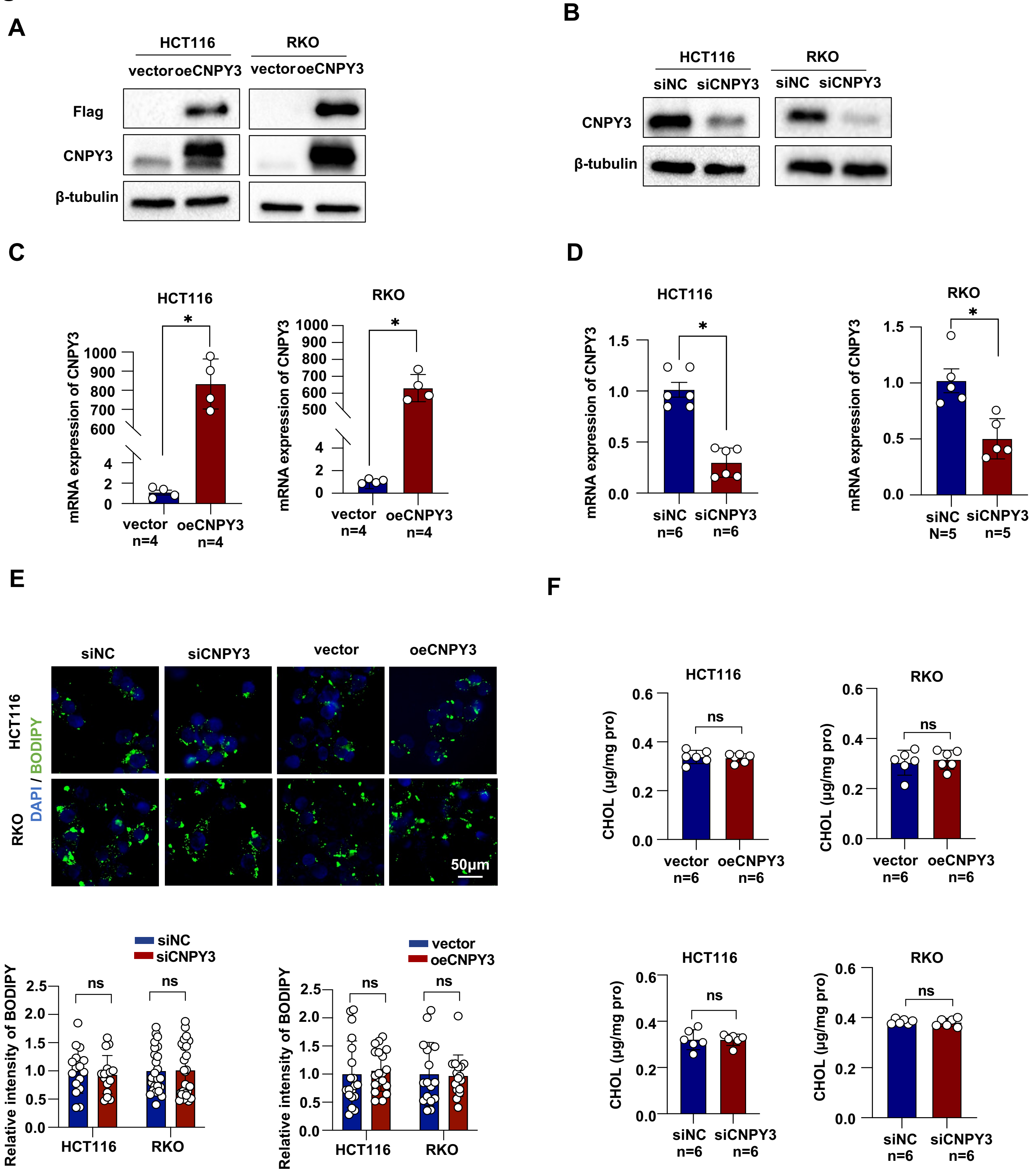
A
HCT116
RKO
vector
oeCNPY3
Flag
CNPY3
β-tubulin
B
HCT116
RKO
siNC
siCNPY3
CNPY3
β-tubulin
C
HCT116
RKO
mRNA expression of CNPY3
vector n=4
oeCNPY3 n=4
D
HCT116
RKO
mRNA expression of CNPY3
siNC n=6
siCNPY3 n=6
siNC N=5
siCNPY3 n=5
E
siNC
siCNPY3
vector
oeCNPY3
HCT116
RKO
DAPI / BODIPY
50μm
Relative intensity of BODIPY
ns
F
HCT116
RKO
CHOL (μg/mg pro)
ns
vector n=6
oeCNPY3 n=6
siNC n=6
siCNPY3 n=6

Figure S6

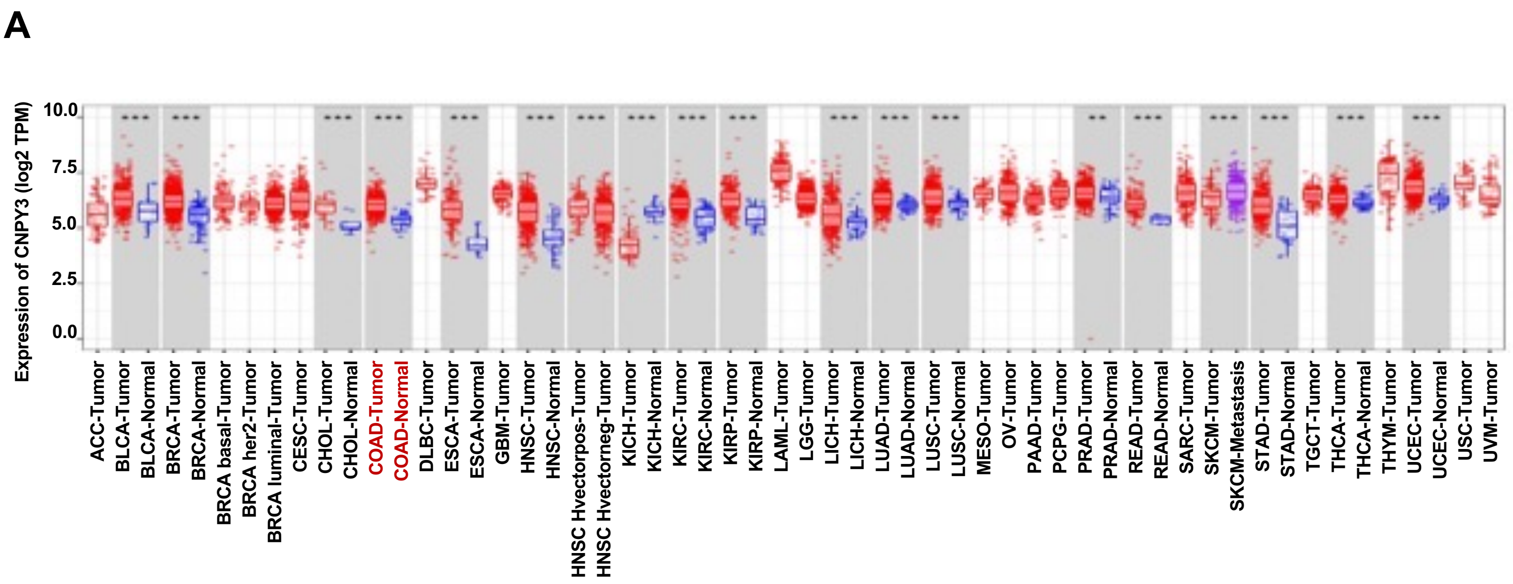
A
Expression of CNPY3 (log2 TPM)
ACC-Tumor
BLCA-Tumor
BLCA-Normal
BRCA-Tumor
BRCA-Normal
BRCA basal-Tumor
BRCA her2-Tumor
BRCA luminal-Tumor
CESC-Tumor
CHOL-Tumor
CHOL-Normal
COAD-Tumor
COAD-Normal
DLBC-Tumor
ESCA-Tumor
ESCA-Normal
GBM-Tumor
HNSC-Tumor
HNSC-Normal
HNSC Hvectorpos-Tumor
HNSC Hvectorneg-Tumor
KICH-Tumor
KICH-Normal
KIRC-Tumor
KIRC-Normal
KIRP-Tumor
KIRP-Normal
LAML-Tumor
LGG-Tumor
LICH-Tumor
LICH-Normal
LUAD-Tumor
LUAD-Normal
LUSC-Tumor
LUSC-Normal
MESO-Tumor
OV-Tumor
PAAD-Tumor
PCPG-Tumor
PRAD-Tumor
PRAD-Normal
READ-Tumor
READ-Normal
SARC-Tumor
SKCM-Tumor
SKCM-Metastasis
STAD-Tumor
STAD-Normal
TGCT-Tumor
THCA-Tumor
THCA-Normal
THYM-Tumor
UCEC-Tumor
UCEC-Normal
USC-Tumor
UVM-Tumor

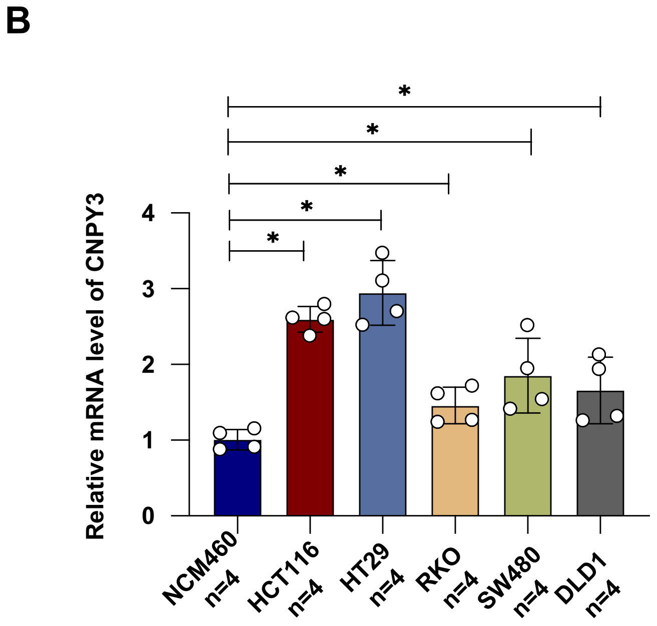
B
Relative mRNA level of CNPY3
NCM460 n=4
HCT116 n=4
HT29 n=4
RKO n=4
SW480 n=4
DLD1 n=4

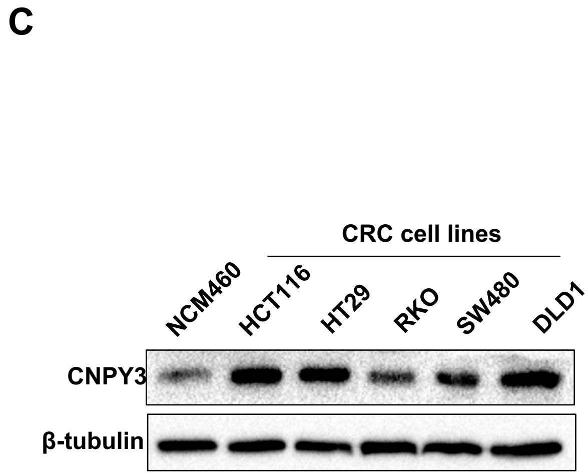
C
CRC cell lines
NCM460
HCT116
HT29
RKO
SW480
DLD1
CNPY3
β-tubulin

Figure S7

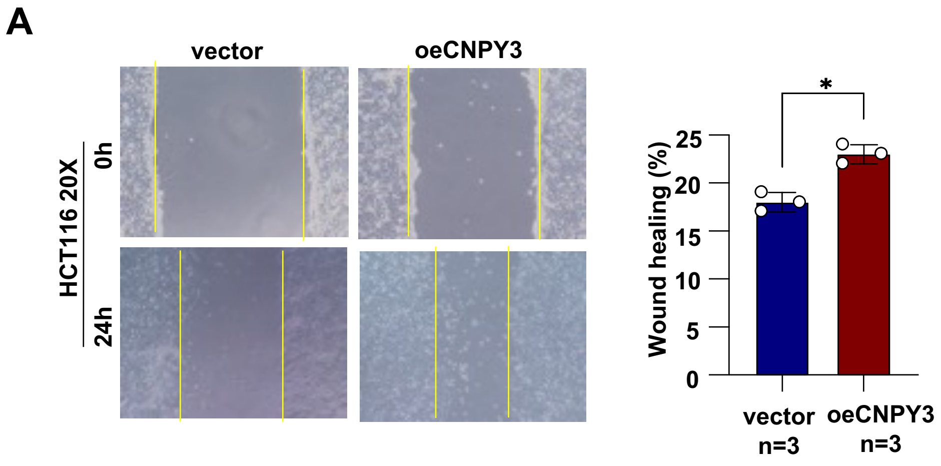
A
vector
oeCNPY3
HCT116 20X
0h
24h
*
Wound healing (%)
25
20
15
10
5
0
vector
n=3
oeCNPY3
n=3

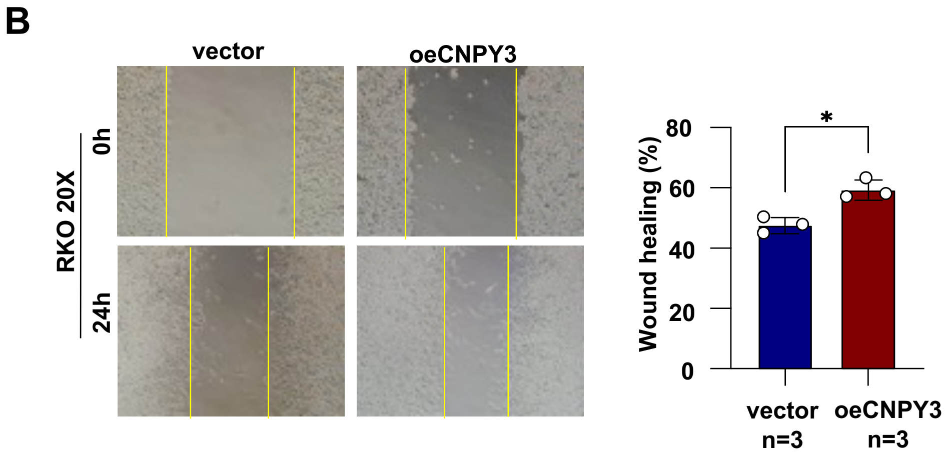
B
vector
oeCNPY3
RKO 20X
0h
24h
*
Wound healing (%)
80
60
40
20
0
vector
n=3
oeCNPY3
n=3

Figure S8

A

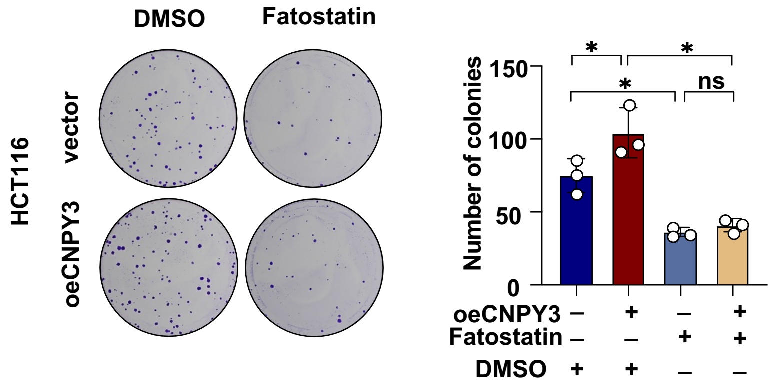
DMSO
Fatostatin
HCT116
vector
oeCNPY3
Number of colonies
150
100
50
0
*
*
*
ns
oeCNPY3 – + – +
Fatostatin – – + +
DMSO + + – –

B

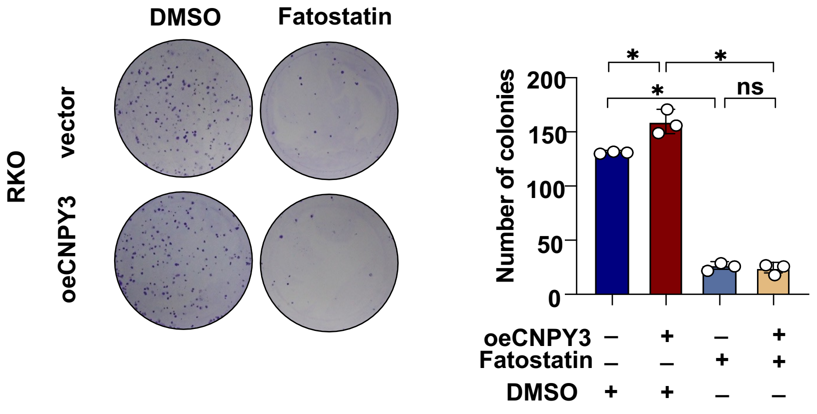
DMSO
Fatostatin
RKO
vector
oeCNPY3
Number of colonies
200
150
100
50
0
*
*
*
ns
oeCNPY3 – + – +
Fatostatin – – + +
DMSO + + – –

Figure S9

A

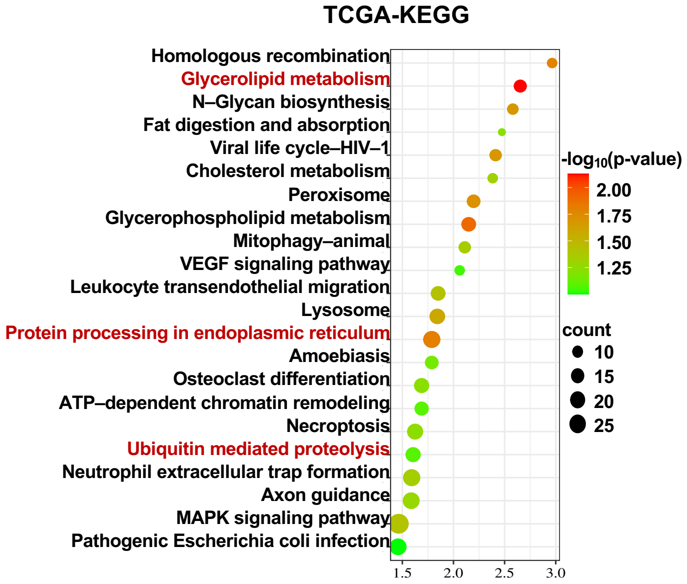
TCGA-KEGG
Homologous recombination
Glycerolipid metabolism
N–Glycan biosynthesis
Fat digestion and absorption
Viral life cycle–HIV–1
Cholesterol metabolism
Peroxisome
Glycerophospholipid metabolism
Mitophagy–animal
VEGF signaling pathway
Leukocyte transendothelial migration
Lysosome
Protein processing in endoplasmic reticulum
Amoebiasis
Osteoclast differentiation
ATP–dependent chromatin remodeling
Necroptosis
Ubiquitin mediated proteolysis
Neutrophil extracellular trap formation
Axon guidance
MAPK signaling pathway
Pathogenic Escherichia coli infection
1.5
2.0
2.5
3.0
-log10(p-value)
2.00
1.75
1.50
1.25
count
10
15
20
25

B

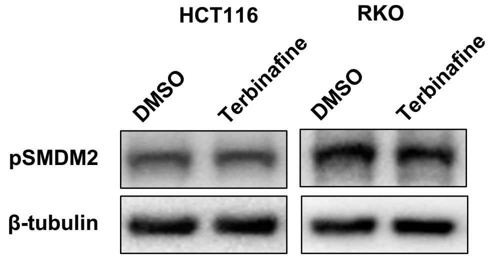
HCT116
RKO
DMSO
Terbinafine
DMSO
Terbinafine
pSMDM2
β-tubulin

Figure S10

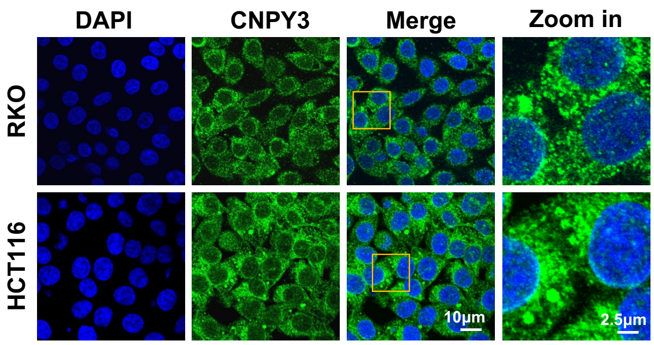

Figure S11

A

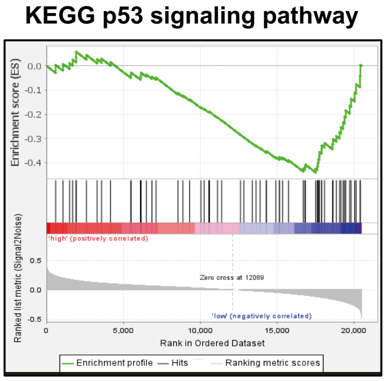

B

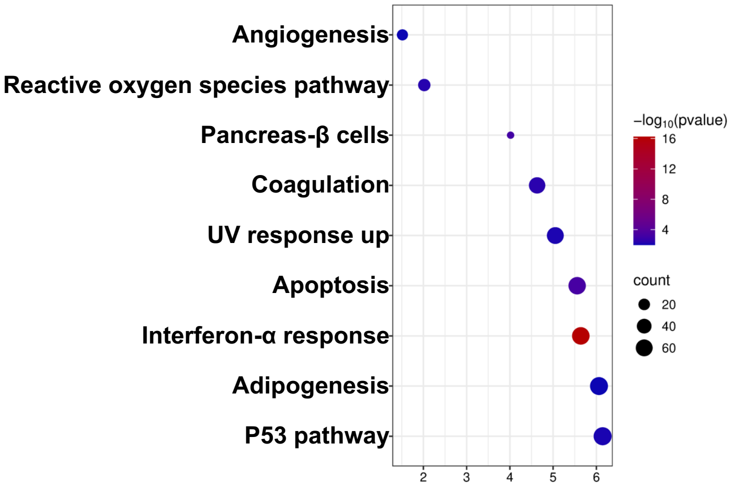

Figure S12

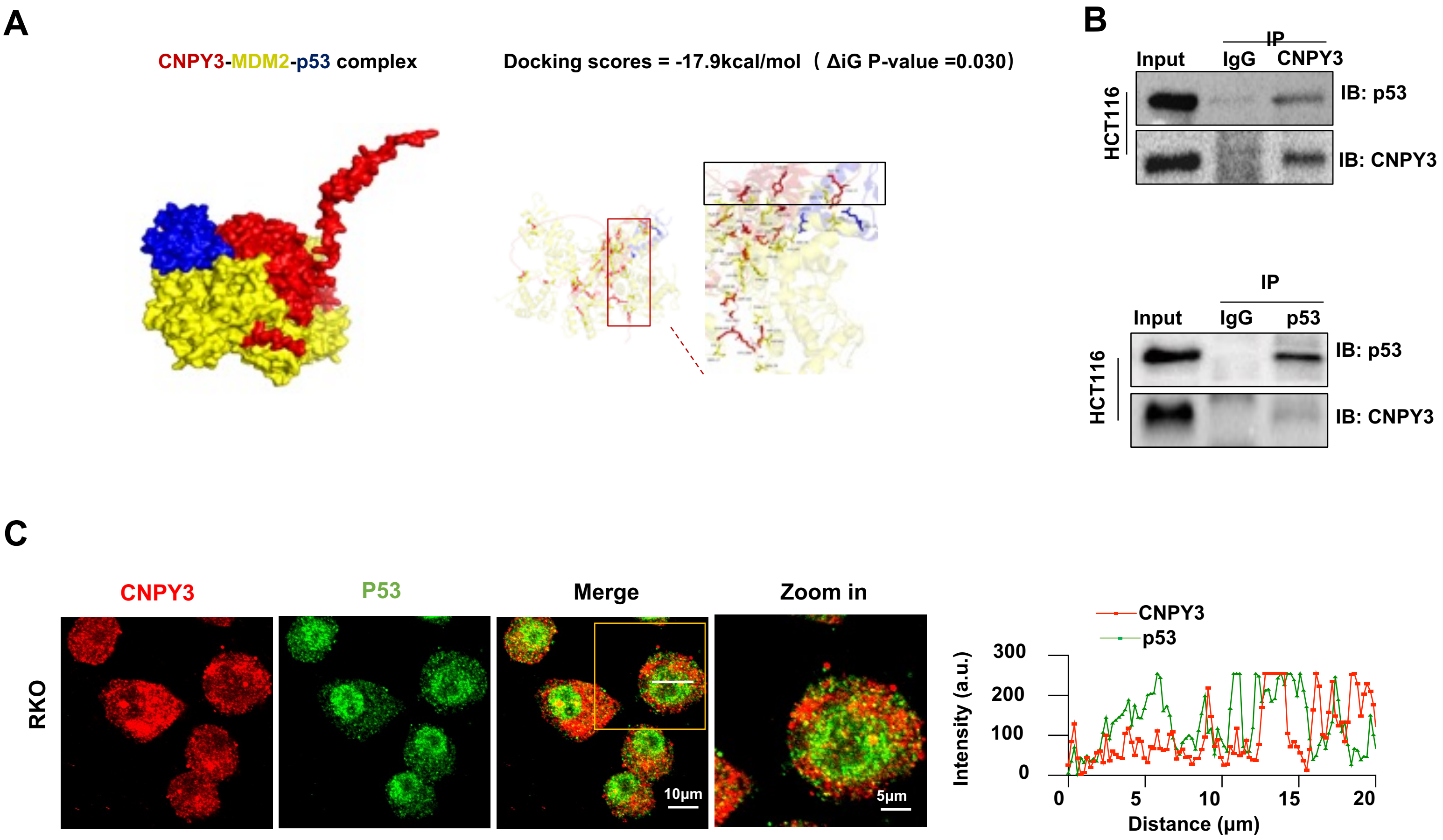
A
CNPY3-MDM2-p53 complex
Docking scores = -17.9kcal/mol （ΔiG P-value =0.030）
B
IP
Input
IgG
CNPY3
HCT116
IB: p53
IB: CNPY3
IP
Input
IgG
p53
HCT116
IB: p53
IB: CNPY3
C
CNPY3
P53
Merge
Zoom in
RKO
10μm
5μm
CNPY3
p53
Intensity (a.u.)
300
200
100
0
0
5
10
15
20
Distance (μm)

Figure S13

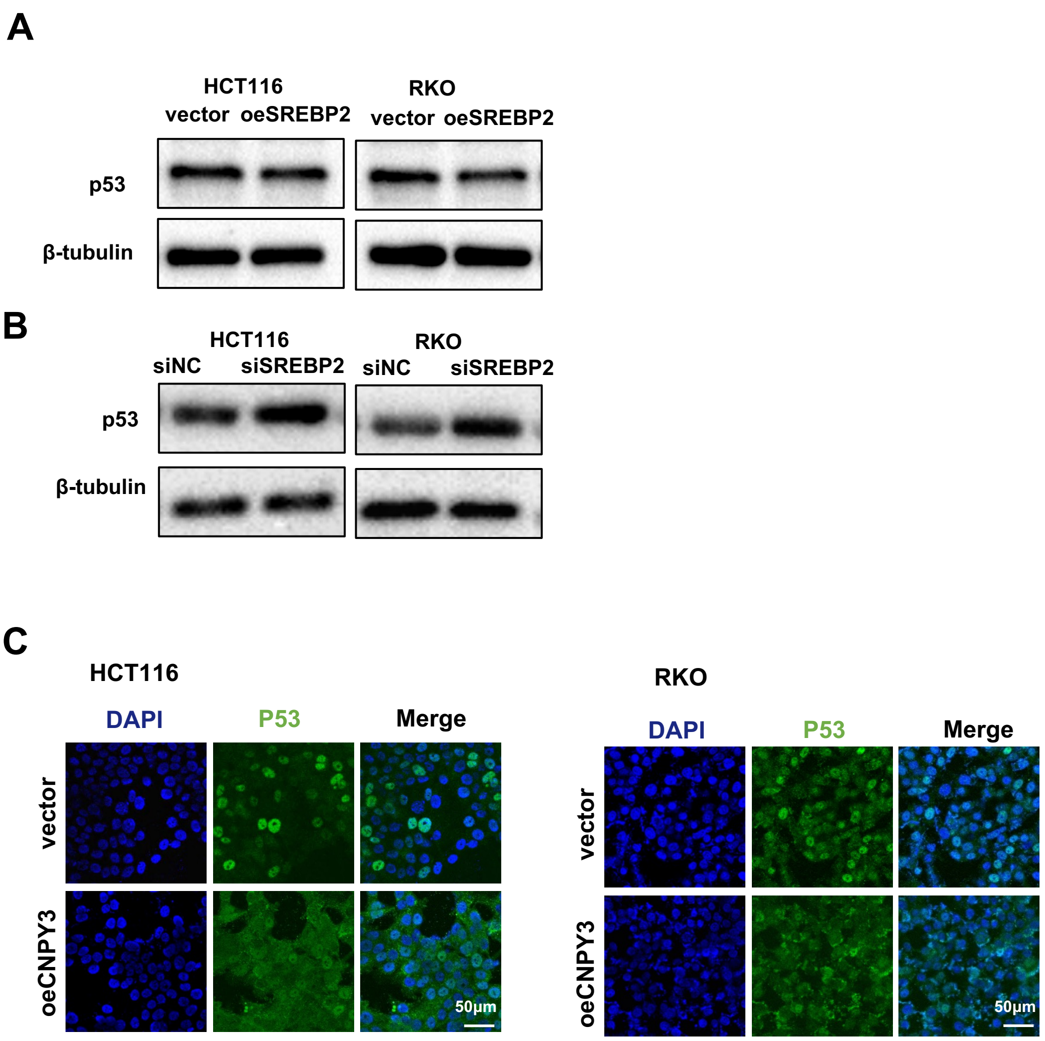
A
HCT116
vector
oeSREBP2
RKO
vector
oeSREBP2
p53
β-tubulin
B
HCT116
siNC
siSREBP2
RKO
siNC
siSREBP2
p53
β-tubulin
C
HCT116
DAPI
P53
Merge
vector
oeCNPY3
50μm
RKO
DAPI
P53
Merge
vector
oeCNPY3
50μm

Figure S14

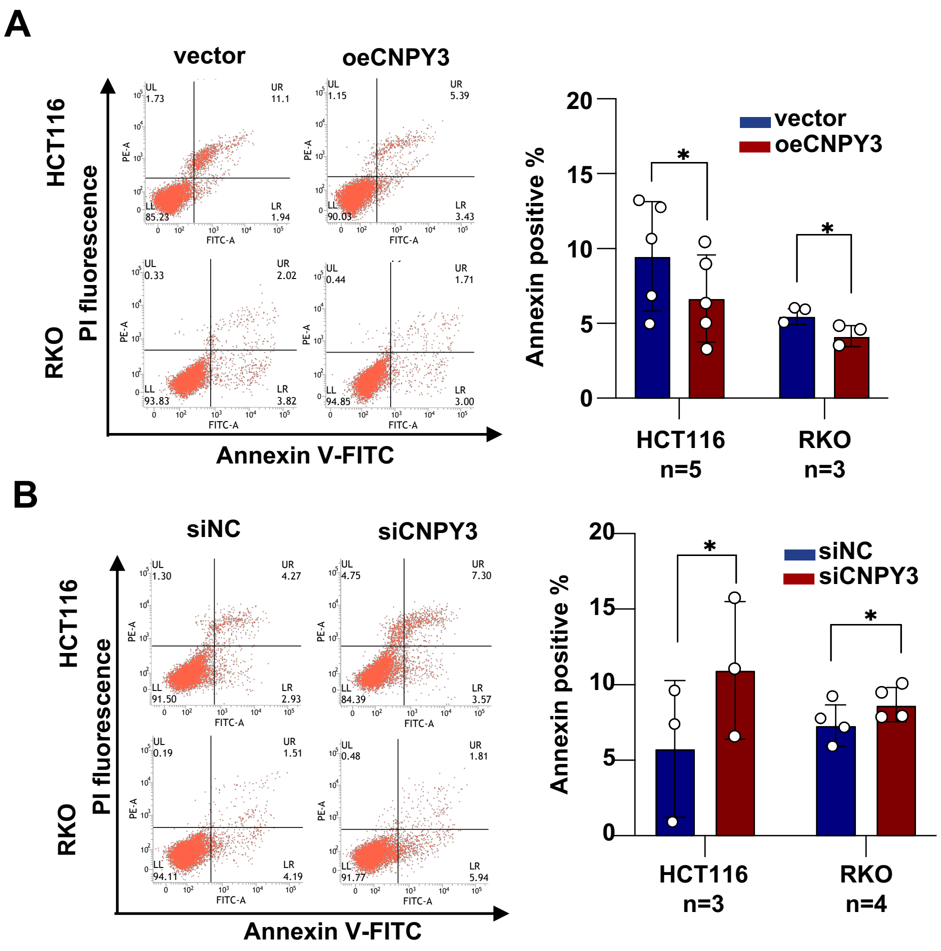
A
vector
oeCNPY3
HCT116
RKO
PI fluorescence
Annexin V-FITC
Annexin positive %
HCT116
n=5
RKO
n=3
B
siNC
siCNPY3
HCT116
RKO
PI fluorescence
Annexin V-FITC
Annexin positive %
HCT116
n=3
RKO
n=4

Figure S15

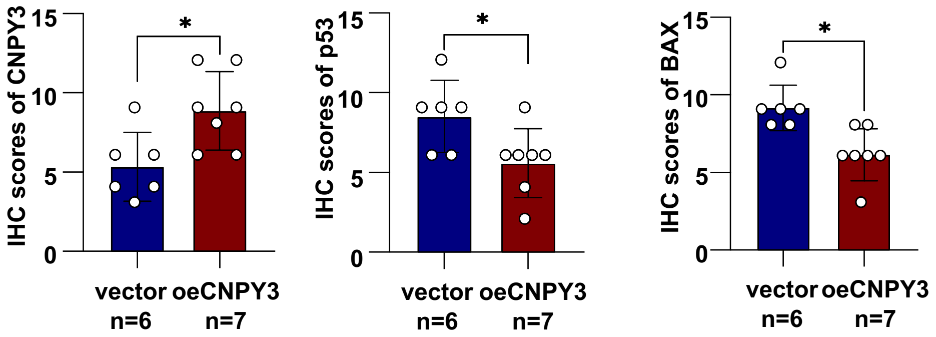
IHC scores of CNPY3
15
10
5
0
*
vector
n=6
oeCNPY3
n=7
IHC scores of p53
15
10
5
0
*
vector
n=6
oeCNPY3
n=7
IHC scores of BAX
15
10
5
0
*
vector
n=6
oeCNPY3
n=7

Figure S16

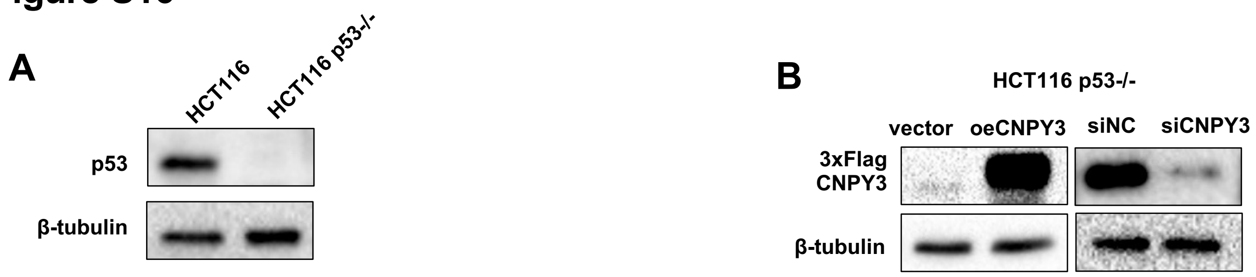
A
HCT116
HCT116 p53-/-
p53
β-tubulin
B
HCT116 p53-/-
vector
oeCNPY3
siNC
siCNPY3
3xFlag
CNPY3
β-tubulin

Figure S17

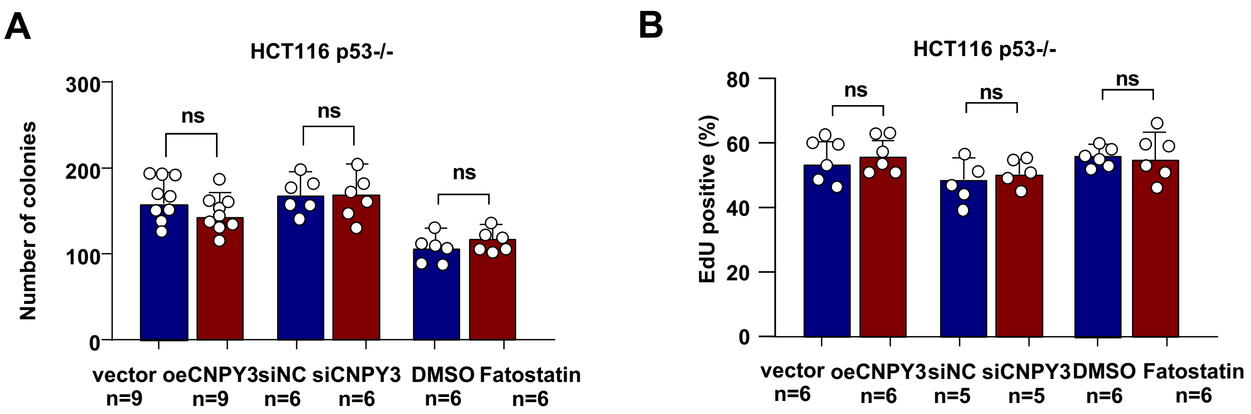
A
HCT116 p53-/-
Number of colonies
300
200
100
0
ns
ns
ns
vector n=9
oeCNPY3 n=9
siNC n=6
siCNPY3 n=6
DMSO n=6
Fatostatin n=6
B
HCT116 p53-/-
EdU positive (%)
80
60
40
20
0
ns
ns
ns
vector n=6
oeCNPY3 n=6
siNC n=5
siCNPY3 n=5
DMSO n=6
Fatostatin n=6

Figure S18

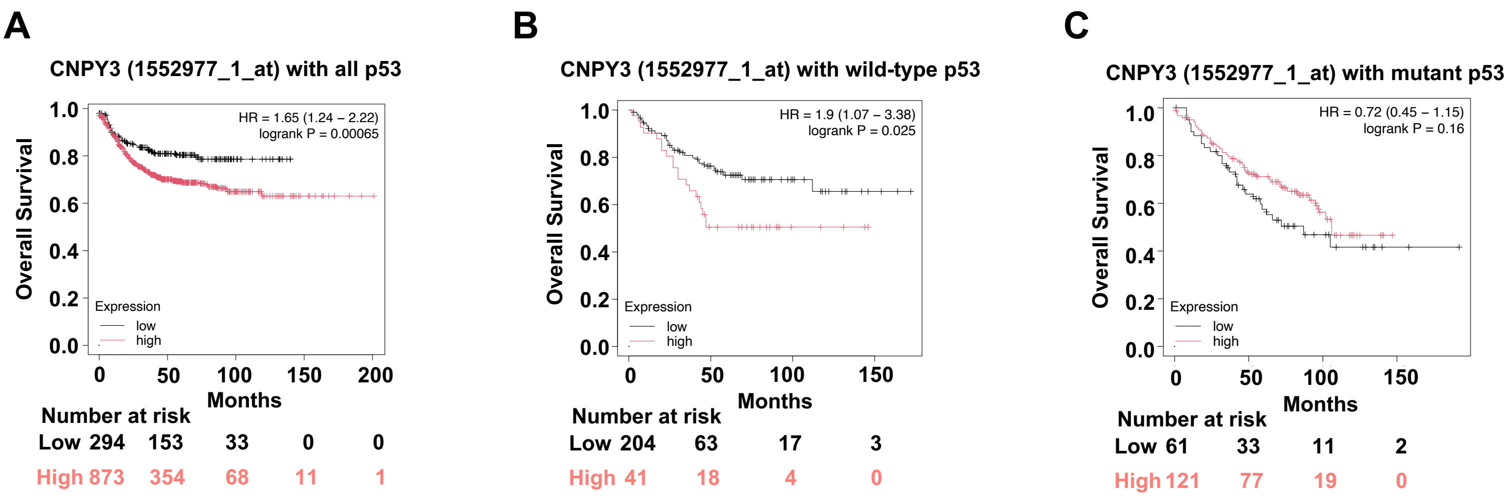
A
CNPY3 (1552977_1_at) with all p53
HR = 1.65 (1.24 – 2.22)
logrank P = 0.00065
Overall Survival
Expression
low
high
Months
Number at risk
Low 294 153 33 0 0
High 873 354 68 11 1
B
CNPY3 (1552977_1_at) with wild-type p53
HR = 1.9 (1.07 – 3.38)
logrank P = 0.025
Overall Survival
Expression
low
high
Months
Number at risk
Low 204 63 17 3
High 41 18 4 0
C
CNPY3 (1552977_1_at) with mutant p53
HR = 0.72 (0.45 – 1.15)
logrank P = 0.16
Overall Survival
Expression
low
high
Months
Number at risk
Low 61 33 11 2
High 121 77 19 0

Figure S19

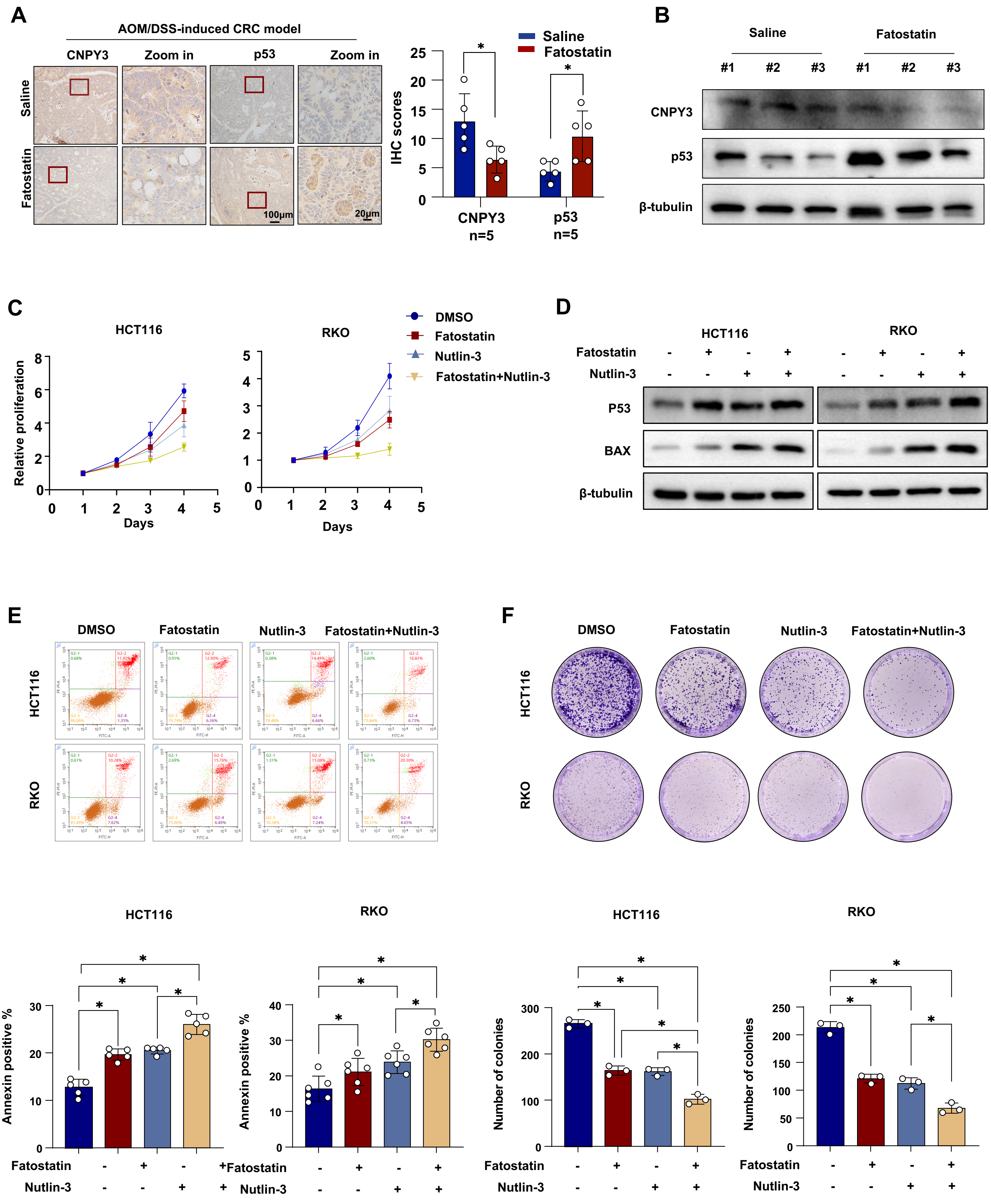
A
AOM/DSS-induced CRC model
CNPY3
Zoom in
p53
Zoom in
Saline
Fatostatin
100μm
20μm
Saline
Fatostatin
IHC scores
CNPY3
n=5
p53
n=5
B
Saline
Fatostatin
#1
#2
#3
#1
#2
#3
CNPY3
p53
β-tubulin
C
HCT116
RKO
DMSO
Fatostatin
Nutlin-3
Fatostatin+Nutlin-3
Relative proliferation
Days
D
HCT116
RKO
Fatostatin
Nutlin-3
P53
BAX
β-tubulin
E
DMSO
Fatostatin
Nutlin-3
Fatostatin+Nutlin-3
HCT116
RKO
F
DMSO
Fatostatin
Nutlin-3
Fatostatin+Nutlin-3
HCT116
RKO
HCT116
RKO
HCT116
RKO
Annexin positive %
Number of colonies
Fatostatin
Nutlin-3

Table S1. The antibodies were applicated in this study.

Antibody name	Company	Catalog No.	Applications
Anti-SREBP2	Abmart	TD7601	IHC (1:100)
Anti-CNPY3	Proteintech	15215-1-AP	WB (1:1000); IHC (1:100); IF (1:100)
Anti-E-cadherin	Cell Signaling Technology	3195	WB (1:1000)
Anti-β-catenin	Cell Signaling Technology	9526	WB (1:1000)
Anti-PCNA	Proteintech	10205-2-AP	WB (1:1000)
Anti-β-tubulin	Proteintech	66240-1-Ig	WB (1:20000)
Anti-SREBP2	Novus	AF7119	ChIP (2 μg per 0.5 ml of cell lysate); WB (1:500)
Anti-PRAT4a (F-6)	Santa Cruz Biotechnology	sc-515151	Co-IP (2 μg per 1 ml of cell lysate)
Anti-mouse IgG	Santa Cruz Biotechnology	sc-2025	Co-IP (2 μg per 1 ml of cell lysate)
Anti-MDM2(SMP14)	Santa Cruz Biotechnology	sc-965	WB (1:100); IF (1:50); Co-IP (2 μg per 1 ml of cell lysate)
Anti-MDM2	Proteintech	66511-1-Ig	WB (1:1000)
Anti-MDM2 (phospho S166)	Abcam	ab170880	WB (1:50000)
Anti-Flag (DDDDK tag)	Abcam	ab205606	WB (1:2000)
Anti-HA tag	ZSGB	CB051	WB (1:1000)
Anti-His tag	Abmart	M20020S	WB (1:1000)
Anti-GST tag	Abcam	AB58626	WB (1:1000)
Anti-p21	Cell Signaling Technology	2947	WB (1:1000)
Anti-p53	Proteintech	10442-1-AP	WB (1:5000); IHC (1:100)
Anti-BAX	Proteintech	50599-2-Ig	WB (1:2000); IHC

			(1:1000); IF (1:100)
Anti-Bcl2	Proteintech	68103-1-Ig	WB (1:500)
Anti-Ki67	Proteintech	27309-1-AP	IHC (1:5000)
Anti-p53 (DO-1)	Santa Cruz Biotechnology	sc-126	IF (1:100); Co-IP (2 μg per 1 ml of cell lysate)
Anti-ubiquitin	Abcam	ab134953	WB (1:1000)
Anti-Rabbit IgG HRP	Affinity	S0001	WB (1:20000)
Anti-Mouse IgG HRP	Affinity	S0002	WB (1:20000)

Note: WB: western blotting; IHC: immunohistochemistry; Co-IP: Co-immunoprecipitation; IF: immunofluorescence staining.

Table S2. Primers were verified and used.

Gene	Forward primer	Reverse primer
CNPY3	5’-CGGAGCTGAGGAGAACGAC-3’	5’-ATAGCCCGTGCCAATCACC-3’
p53	5’-CAGCACATGACGGAGGTTGT-3’	5’-CAGCACATGACGGAGGTTGT-3’
GAPDH	5’-GGAGCGAGATCCCTCCAAAAT-3’	5’-GGCTGTTGTCATACTTCTCATGG-3’
MDM2	5’-CAGTAGCAGTGAATCTACAGGGA-3’	5’-CTGATCCAACCAATCACCTGAAT-3’
CNPY3 pro-1	5’-GGACTTTGCCATAGGTCGCT-3’	5’-AGCCAATAGGCTTTCTGGGG-3’
CNPY3 pro-2	5’-GCCCGGCTATAACAACCACA-3’	5’-GTCCTTTCCCACCCCCTAAC-3’

Table S3. The two clusters of TCGA-COAD population.

Group 1	Group 2
TCGA-CA-6718-01A-11R-1839-07	TCGA-AA-3549-01A-02R-0821-07
TCGA-AD-6901-01A-11R-1928-07	TCGA-G4-6626-01A-11R-1774-07
TCGA-AA-A00J-01A-02R-A002-07	TCGA-AA-3973-01A-01R-1022-07
TCGA-CM-4752-01A-01R-1410-07	TCGA-AA-3679-01A-02R-0905-07
TCGA-AA-3966-01A-01R-1113-07	TCGA-A6-4107-01A-02R-1410-07
TCGA-AA-3856-01A-01R-0905-07	TCGA-CK-5915-01A-11R-1653-07
TCGA-AZ-4615-01A-01R-1410-07	TCGA-CK-6751-01A-11R-1839-07
TCGA-A6-A566-01A-11R-A28H-07	TCGA-AA-3955-01A-02R-1022-07
TCGA-AA-3877-01A-01R-1022-07	TCGA-AA-3855-01A-01R-1022-07
TCGA-A6-2674-01A-02R-0821-07	TCGA-F4-6856-01A-11R-1928-07
TCGA-AZ-4308-01A-01R-1410-07	TCGA-A6-5659-01B-04R-A277-07
TCGA-AA-A00K-01A-02R-A002-07	TCGA-DM-A280-01A-12R-A16W-07
TCGA-AA-A01P-01A-21R-A083-07	TCGA-A6-2674-01A-02R-A278-07
TCGA-AA-3548-01A-01R-1873-07	TCGA-AA-A00O-01A-02R-A089-07
TCGA-G4-6297-01A-11R-1723-07	TCGA-QG-A5YV-01A-11R-A28H-07
TCGA-AA-3982-01A-02R-1022-07	TCGA-AA-A01V-01A-23R-A083-07
TCGA-AA-3875-01A-01R-0905-07	TCGA-G4-6320-01A-11R-1723-07
TCGA-A6-2671-01A-01R-1410-07	TCGA-DM-A288-01A-11R-A16W-07
TCGA-AA-3977-01A-01R-1022-07	TCGA-NH-A6GA-01A-11R-A37K-07
TCGA-D5-6929-01A-31R-1928-07	TCGA-D5-5540-01A-01R-1653-07
TCGA-AM-5821-01A-01R-1653-07	TCGA-CA-5797-01A-01R-1653-07
TCGA-AZ-6605-01A-11R-1839-07	TCGA-CM-6161-01A-11R-1653-07
TCGA-A6-6780-01A-11R-1839-07	TCGA-AA-3509-01A-01R-1410-07
TCGA-CK-6746-01A-11R-1839-07	TCGA-AA-3502-01A-01R-1410-07
TCGA-CM-5348-01A-21R-1723-07	TCGA-A6-6650-01A-11R-1774-07
TCGA-A6-3807-01A-01R-1022-07	TCGA-AA-A01G-01A-01R-A002-07
TCGA-AA-A01R-01A-21R-A083-07	TCGA-AA-A01I-01A-02R-A089-07
TCGA-AA-3812-01A-01R-0905-07	TCGA-DM-A0XD-01A-12R-A155-07
TCGA-A6-3808-01A-01R-1022-07	TCGA-D5-6532-01A-11R-1723-07
TCGA-A6-2672-01A-01R-0826-07	TCGA-DM-A28E-01A-11R-A32Y-07
TCGA-CM-6168-01A-11R-1653-07	TCGA-A6-6780-01B-04R-A277-07
TCGA-F4-6855-01A-11R-1928-07	TCGA-CM-6163-01A-11R-1653-07
TCGA-AA-3492-01A-01R-1410-07	TCGA-AA-A01Z-01A-11R-A083-07
TCGA-WS-AB45-01A-11R-A41B-07	TCGA-D5-7000-01A-11R-A32Z-07
TCGA-AA-A02H-01A-01R-A089-07	TCGA-CM-4743-01A-01R-1723-07
TCGA-AA-3553-01A-01R-0821-07	TCGA-CK-4947-01B-11R-1653-07
TCGA-CM-6167-01A-11R-1653-07	TCGA-A6-A567-01A-31R-A28H-07
TCGA-A6-5664-01A-21R-1839-07	TCGA-QL-A97D-01A-12R-A41B-07
TCGA-D5-6928-01A-11R-1928-07	TCGA-A6-2672-01B-03R-2302-07
TCGA-D5-6530-01A-11R-1723-07	TCGA-D5-6540-01A-11R-1723-07
TCGA-A6-2685-01A-01R-1410-07	TCGA-AA-3845-01A-01R-1022-07
TCGA-AA-3994-01A-01R-1113-07	TCGA-A6-6137-01A-11R-1774-07
TCGA-AA-3837-01A-01R-0905-07	TCGA-DM-A1HB-01A-21R-A180-07
TCGA-G4-6299-01A-11R-1774-07	TCGA-AA-3519-01A-02R-0821-07
TCGA-AZ-6600-01A-11R-1774-07	TCGA-CM-6172-01A-11R-1653-07

TCGA-AY-6196-01A-11R-1723-07
TCGA-AA-A00E-01A-01R-A002-07
TCGA-CK-5916-01A-11R-1653-07
TCGA-A6-A5ZU-01A-11R-A28H-07
TCGA-A6-A56B-01A-31R-A28H-07
TCGA-AA-3831-01A-01R-0905-07
TCGA-D5-6924-01A-11R-1928-07
TCGA-CK-6748-01A-11R-1839-07
TCGA-AA-3489-01A-21R-1839-07
TCGA-F4-6805-01A-11R-1839-07
TCGA-A6-5657-01A-01R-A32Z-07
TCGA-NH-A50V-01A-11R-A28H-07
TCGA-A6-3810-01A-01R-A278-07
TCGA-AD-6899-01A-11R-1928-07
TCGA-D5-5539-01A-01R-1653-07
TCGA-D5-6930-01A-11R-1928-07
TCGA-NH-A8F8-01A-72R-A41B-07
TCGA-A6-2675-01A-02R-1723-07
TCGA-D5-6541-01A-11R-1723-07
TCGA-AA-3869-01A-01R-1022-07
TCGA-AM-5820-01A-01R-1653-07
TCGA-CK-4948-01B-11R-1653-07
TCGA-AA-3852-01A-01R-0905-07
TCGA-G4-6302-01A-11R-1723-07
TCGA-A6-6780-01A-11R-A278-07
TCGA-AA-A02R-01A-01R-A00A-07
TCGA-AA-A017-01A-01R-A00A-07
TCGA-D5-5541-01A-01R-1653-07
TCGA-AA-3989-01A-01R-1022-07
TCGA-D5-6531-01A-11R-1723-07
TCGA-AA-3939-01A-01R-1022-07
TCGA-NH-A5IV-01A-42R-A37K-07
TCGA-A6-2680-01A-01R-1410-07
TCGA-D5-5538-01A-01R-1653-07
TCGA-AA-3510-01A-01R-1410-07
TCGA-F4-6809-01A-11R-1839-07
TCGA-AA-A03F-01A-11R-A16W-07
TCGA-AA-3552-01A-01R-0821-07
TCGA-AA-3867-01A-01R-1022-07
TCGA-AD-6895-01A-11R-1928-07
TCGA-AA-A01D-01A-01R-A00A-07
TCGA-AA-A01C-01A-01R-A00A-07
TCGA-DM-A28A-01A-21R-A32Y-07
TCGA-AA-3521-01A-01R-0821-07
TCGA-A6-6782-01A-11R-1839-07
TCGA-AD-6890-01A-11R-1928-07
TCGA-D5-6534-01A-21R-1928-07
TCGA-CM-6169-01A-11R-1653-07
TCGA-DM-A1HA-01A-11R-A155-07
TCGA-CA-5796-01A-01R-1653-07
TCGA-AA-A00F-01A-01R-A002-07
TCGA-A6-6650-01B-02R-A277-07
TCGA-AY-A69D-01A-11R-A37K-07
TCGA-CK-6747-01A-11R-1839-07
TCGA-CM-6678-01A-11R-1839-07
TCGA-AD-A5EK-01A-11R-A28H-07
TCGA-DM-A1D6-01A-21R-A155-07
TCGA-AY-A54L-01A-11R-A28H-07
TCGA-DM-A1D9-01A-11R-A155-07
TCGA-CM-6165-01A-11R-1653-07
TCGA-A6-5656-01B-02R-A277-07
TCGA-AA-3660-01A-01R-1723-07
TCGA-D5-6533-01A-11R-1723-07
TCGA-AA-A00W-01A-01R-A002-07
TCGA-DM-A1D7-01A-11R-A155-07
TCGA-D5-6536-01A-11R-1723-07
TCGA-AU-3779-01A-01R-1723-07
TCGA-AZ-6606-01A-11R-1839-07
TCGA-AA-3956-01A-02R-1022-07
TCGA-AA-A02J-01A-01R-A00A-07
TCGA-A6-2684-01C-08R-A277-07
TCGA-F4-6854-01A-11R-1928-07
TCGA-AY-5543-01A-01R-1653-07
TCGA-F4-6808-01A-11R-1839-07
TCGA-AA-3495-01A-01R-1410-07
TCGA-5M-AAT5-01A-21R-A41B-07
TCGA-F4-6460-01A-11R-1774-07
TCGA-D5-6931-01A-11R-1928-07
TCGA-AA-3494-01A-01R-1410-07
TCGA-NH-A50T-01A-11R-A28H-07
TCGA-A6-3809-01B-04R-A277-07
TCGA-A6-5662-01A-01R-1653-07
TCGA-CM-4746-01A-01R-1410-07
TCGA-AA-A02Y-01A-43R-A32Y-07
TCGA-4N-A93T-01A-11R-A37K-07
TCGA-AA-3678-01A-01R-0905-07
TCGA-CM-4744-01A-01R-A32Z-07
TCGA-AA-3534-01A-01R-0821-07
TCGA-AY-6197-01A-11R-1723-07
TCGA-DM-A28G-01A-11R-A16W-07
TCGA-AA-3529-01A-02R-0821-07
TCGA-A6-3810-01B-04R-A277-07
TCGA-CM-6675-01A-11R-1839-07
TCGA-AA-A00U-01A-01R-A002-07
TCGA-CA-6715-01A-21R-1839-07
TCGA-D5-6923-01A-11R-A32Z-07

TCGA-AA-3562-01A-02R-0821-07
TCGA-AD-6548-01A-11R-1839-07
TCGA-AD-6964-01A-11R-1928-07
TCGA-AA-3516-01A-02R-0826-07
TCGA-CM-4750-01A-01R-1410-07
TCGA-AA-3554-01A-01R-0826-07
TCGA-A6-2676-01A-01R-0826-07
TCGA-AA-3986-01A-02R-1022-07
TCGA-CM-5860-01A-01R-1653-07
TCGA-A6-6654-01A-21R-1839-07
TCGA-AA-3975-01A-01R-1022-07
TCGA-AA-3692-01A-01R-0905-07
TCGA-AA-3506-01A-01R-1410-07
TCGA-AA-3543-01A-01R-0826-07
TCGA-A6-2682-01A-01R-1410-07
TCGA-AA-A00D-01A-01R-A002-07
TCGA-5M-AAT6-01A-11R-A41B-07
TCGA-AA-3862-01A-01R-1022-07
TCGA-CM-6162-01A-11R-1653-07
TCGA-AA-3872-01A-01R-1022-07
TCGA-CM-5349-01A-21R-1723-07
TCGA-AA-3526-01A-02R-A32Z-07
TCGA-AA-3866-01A-01R-1022-07
TCGA-AA-A022-01A-21R-A16W-07
TCGA-A6-2681-01A-01R-1410-07
TCGA-G4-6311-01A-11R-1723-07
TCGA-AZ-4684-01A-01R-1410-07
TCGA-AA-3544-01A-01R-1873-07
TCGA-F4-6570-01A-11R-1774-07
TCGA-AA-3970-01A-01R-1022-07
TCGA-AA-3842-01A-01R-1022-07
TCGA-AZ-4315-01A-01R-1410-07
TCGA-A6-6781-01A-22R-A278-07
TCGA-AZ-4616-01A-21R-1839-07
TCGA-F4-6703-01A-11R-1839-07
TCGA-CM-5344-01A-21R-1723-07
TCGA-D5-6529-01A-11R-1774-07
TCGA-CM-6674-01A-11R-1839-07
TCGA-CM-5341-01A-01R-1410-07
TCGA-AA-3870-01A-01R-1022-07
TCGA-AA-3858-01A-01R-0905-07
TCGA-AZ-6601-01A-11R-1774-07
TCGA-A6-4105-01A-02R-1774-07
TCGA-AA-3814-01A-01R-0905-07
TCGA-AA-3527-01A-01R-0821-07
TCGA-AA-3672-01A-01R-0905-07
TCGA-AA-3811-01A-01R-1022-07
TCGA-AA-3715-01A-01R-0905-07
TCGA-AA-3673-01A-01R-0905-07
TCGA-CM-6166-01A-11R-1653-07
TCGA-A6-5659-01A-01R-1653-07
TCGA-A6-5661-01B-05R-2302-07
TCGA-G4-6309-01A-21R-1839-07
TCGA-A6-2674-01B-04R-A277-07
TCGA-DM-A28F-01A-11R-A32Y-07
TCGA-AA-A02E-01A-01R-A00A-07
TCGA-5M-AAT4-01A-11R-A41B-07
TCGA-DM-A1D0-01A-11R-A155-07
TCGA-G4-6293-01A-11R-1723-07
TCGA-AD-6963-01A-11R-1928-07
TCGA-RU-A8FL-01A-11R-A37K-07
TCGA-AA-3941-01A-01R-1022-07
TCGA-DM-A1D4-01A-21R-A155-07
TCGA-AA-3681-01A-01R-0905-07
TCGA-4T-AA8H-01A-11R-A41B-07
TCGA-F4-6806-01A-11R-1839-07
TCGA-F4-6459-01A-11R-1774-07
TCGA-G4-6322-01A-11R-1723-07
TCGA-5M-AATE-01A-11R-A41B-07
TCGA-AZ-5407-01A-01R-1723-07
TCGA-AA-3696-01A-01R-0905-07
TCGA-DM-A1DA-01A-11R-A155-07
TCGA-A6-5656-01A-21R-1839-07
TCGA-AA-3685-01A-02R-A32Z-07
TCGA-A6-A565-01A-31R-A28H-07
TCGA-AA-3675-01A-02R-0905-07
TCGA-AA-3511-01A-21R-1839-07
TCGA-AA-3971-01A-01R-1022-07
TCGA-DM-A28K-01A-21R-A32Y-07
TCGA-AA-A02O-01A-21R-A16W-07
TCGA-CM-6676-01A-11R-1839-07
TCGA-AD-A5EJ-01A-11R-A28H-07
TCGA-AA-3560-01A-01R-0821-07
TCGA-CM-6680-01A-11R-1839-07
TCGA-G4-6627-01A-11R-1774-07
TCGA-AA-3861-01A-01R-1022-07
TCGA-AA-3833-01A-01R-0905-07
TCGA-A6-2677-01A-01R-0821-07
TCGA-A6-2677-01A-01R-A278-07
TCGA-AA-3972-01A-01R-1022-07
TCGA-G4-6314-01A-11R-1723-07
TCGA-G4-6588-01A-11R-1774-07
TCGA-AA-3976-01A-01R-1022-07
TCGA-AA-3979-01A-01R-1022-07
TCGA-AA-3556-01A-01R-0821-07
TCGA-CA-5255-01A-11R-1839-07

TCGA-G4-6625-01A-21R-1774-07
TCGA-G4-6628-01A-11R-1839-07
TCGA-AA-3496-01A-21R-1839-07
TCGA-AA-3950-01A-02R-1022-07
TCGA-G4-6586-01A-11R-1774-07
TCGA-AA-3952-01A-01R-1022-07
TCGA-AA-3662-01A-01R-1723-07
TCGA-A6-6781-01A-22R-1928-07
TCGA-F4-6807-01A-11R-1839-07
TCGA-A6-6138-01A-11R-1774-07
TCGA-AA-A03J-01A-21R-A16W-07
TCGA-AA-A00Q-01A-01R-A002-07
TCGA-AA-3532-01A-01R-0821-07
TCGA-AA-3538-01A-01R-0821-07
TCGA-AA-3815-01A-01R-1022-07
TCGA-D5-6932-01A-11R-1928-07
TCGA-A6-2686-01A-01R-A32Z-07
TCGA-CM-5868-01A-01R-1653-07
TCGA-AZ-4323-01A-21R-1839-07
TCGA-AA-3949-01A-01R-1022-07
TCGA-AA-A004-01A-01R-A00A-07
TCGA-AU-6004-01A-11R-1723-07
TCGA-A6-5660-01A-01R-1653-07
TCGA-AA-3710-01A-01R-1022-07
TCGA-AA-3684-01A-02R-0905-07
TCGA-F4-6704-01A-11R-1839-07
TCGA-A6-2684-01A-01R-A278-07
TCGA-A6-2684-01A-01R-1410-07
TCGA-AA-3930-01A-01R-1022-07
TCGA-CK-4951-01A-01R-1410-07
TCGA-A6-6651-01A-21R-1839-07
TCGA-CK-5913-01A-11R-1653-07
TCGA-D5-6898-01A-11R-1928-07
TCGA-A6-2679-01A-02R-1410-07
TCGA-F4-6461-01A-11R-1774-07
TCGA-A6-6141-01A-11R-1774-07
TCGA-AA-3520-01A-01R-0821-07
TCGA-AY-4070-01A-01R-1113-07
TCGA-CA-6719-01A-11R-1839-07
TCGA-AA-A01K-01A-01R-A00A-07
TCGA-D5-6927-01A-21R-1928-07
TCGA-AA-3517-01A-01R-0821-07
TCGA-AZ-6598-01A-11R-1774-07
TCGA-A6-6142-01A-11R-1774-07
TCGA-F4-6569-01A-11R-1774-07
TCGA-AA-A00N-01A-02R-A00A-07
TCGA-CM-4751-01A-02R-1839-07
TCGA-G4-6304-01A-11R-1928-07
TCGA-AA-3713-01A-21R-1723-07
TCGA-AA-A02K-01A-03R-A32Y-07
TCGA-D5-6920-01A-11R-1928-07
TCGA-G4-6306-01A-11R-1774-07
TCGA-QG-A5Z1-01A-11R-A28H-07
TCGA-AA-3655-01A-02R-1723-07
TCGA-AZ-4681-01A-01R-1410-07
TCGA-A6-6649-01A-11R-1774-07
TCGA-A6-2677-01B-02R-A277-07
TCGA-AA-A00Z-01A-01R-A002-07
TCGA-G4-6307-01A-11R-1723-07
TCGA-AA-3561-01A-01R-0821-07
TCGA-AA-3712-01A-21R-1723-07
TCGA-AY-4071-01A-01R-1113-07
TCGA-CM-5863-01A-21R-1839-07
TCGA-A6-6652-01A-11R-1774-07
TCGA-DM-A28H-01A-11R-A16W-07
TCGA-DM-A1D8-01A-11R-A155-07
TCGA-AA-3819-01A-01R-0905-07
TCGA-A6-2678-01A-01R-0821-07
TCGA-QG-A5YX-01A-11R-A28H-07
TCGA-CK-4952-01A-01R-1723-07
TCGA-AA-3693-01A-01R-0905-07
TCGA-A6-6140-01A-11R-1774-07
TCGA-AA-3821-01A-01R-1022-07
TCGA-AD-6889-01A-11R-1928-07
TCGA-AA-3984-01A-02R-1022-07
TCGA-CM-6164-01A-11R-1653-07
TCGA-CK-5912-01A-11R-1653-07
TCGA-A6-6653-01A-11R-1774-07
TCGA-CK-4950-01A-01R-1723-07
TCGA-AD-6965-01A-11R-1928-07
TCGA-DM-A0XF-01A-11R-A155-07
TCGA-A6-6648-01A-11R-1774-07
TCGA-AA-3844-01A-01R-1022-07
TCGA-CA-5256-01A-01R-1410-07
TCGA-AA-A01X-01A-21R-A083-07
TCGA-AA-3850-01A-01R-1022-07
TCGA-A6-6781-01B-06R-A277-07
TCGA-AA-3697-01A-01R-1723-07
TCGA-AY-A71X-01A-12R-A37K-07
TCGA-AA-A029-01A-01R-A00A-07
TCGA-AZ-4313-01A-01R-1410-07
TCGA-AA-3524-01A-02R-0821-07
TCGA-AA-3860-01A-02R-0905-07
TCGA-G4-6310-01A-11R-1723-07
TCGA-G4-6321-01A-11R-1723-07
TCGA-T9-A92H-01A-11R-A37K-07

TCGA-D5-6926-01A-11R-1928-07
TCGA-CA-6717-01A-11R-1839-07
TCGA-A6-3810-01A-01R-1022-07
TCGA-CA-5254-01A-21R-1839-07
TCGA-QG-A5YW-01A-11R-A28H-07
TCGA-AA-A010-01A-01R-A089-07
TCGA-AD-5900-01A-11R-1653-07
TCGA-AA-A01Q-01A-01R-A002-07
TCGA-AA-A00A-01A-01R-A002-07
TCGA-AZ-6607-01A-11R-1839-07
TCGA-CM-4747-01A-01R-1410-07
TCGA-A6-3809-01A-01R-1022-07
TCGA-NH-A50U-01A-33R-A37K-07
TCGA-CM-6679-01A-11R-1839-07
TCGA-D5-6535-01A-11R-1723-07
TCGA-AA-A00R-01A-01R-A002-07
TCGA-AA-3518-01A-02R-0826-07
TCGA-CM-4748-01A-01R-1410-07
TCGA-G4-6295-01A-11R-1723-07
TCGA-AA-3688-01A-01R-0905-07
TCGA-AA-3530-01A-01R-1022-07
TCGA-AY-A8YK-01A-11R-A41B-07
TCGA-AZ-6599-01A-11R-1774-07
TCGA-CA-6716-01A-11R-1839-07
TCGA-3L-AA1B-01A-11R-A37K-07
TCGA-AA-A024-01A-02R-A00A-07
TCGA-G4-6298-01A-11R-1723-07
TCGA-G4-6294-01A-11R-1774-07
TCGA-DM-A285-01A-11R-A16W-07
TCGA-AA-3522-01A-01R-0821-07
TCGA-AD-6888-01A-11R-1928-07
TCGA-AA-3531-01A-01R-0821-07
TCGA-AA-3525-01A-02R-0826-07
TCGA-AZ-6608-01A-11R-1839-07
TCGA-AA-A00L-01A-01R-A002-07
TCGA-AZ-6603-01A-11R-1839-07
TCGA-CM-6170-01A-11R-1653-07
TCGA-D5-6537-01A-11R-1723-07
TCGA-AA-3695-01A-01R-0905-07
TCGA-AA-3854-01A-01R-0905-07
TCGA-AA-3848-01A-01R-0905-07
TCGA-SS-A7HO-01A-21R-A37K-07
TCGA-AZ-4614-01A-01R-1410-07
TCGA-CM-6171-01A-11R-1653-07
TCGA-AA-3542-01A-02R-1873-07
TCGA-G4-6317-02A-11R-2066-07
TCGA-AA-3980-01A-02R-1022-07
TCGA-AA-3514-01A-02R-0821-07
TCGA-G4-6315-01A-11R-1723-07
TCGA-DM-A28M-01A-12R-A16W-07
TCGA-AA-3667-01A-01R-0905-07
TCGA-AA-3663-01A-01R-1723-07
TCGA-G4-6323-01A-11R-1723-07
TCGA-AA-A01T-01A-21R-A16W-07
TCGA-AA-A01F-01A-01R-A002-07
TCGA-QG-A5Z2-01A-11R-A28H-07
TCGA-NH-A6GC-01A-12R-A41B-07
TCGA-A6-5667-01A-21R-1723-07
TCGA-AA-3851-01A-01R-1022-07
TCGA-NH-A8F7-01A-11R-A41B-07
TCGA-G4-6303-01A-11R-1774-07
TCGA-NH-A8F7-06A-31R-A41B-07
TCGA-A6-6650-01A-11R-A278-07
TCGA-CK-5914-01A-11R-1653-07
TCGA-AA-3488-01A-01R-1410-07
TCGA-CM-5861-01A-01R-1653-07
TCGA-AZ-5403-01A-01R-1653-07

	TCGA-DM-A0X9-01A-11R-A155-07
	TCGA-D5-5537-01A-21R-1928-07
	TCGA-5M-AATA-01A-31R-A41B-07
	TCGA-F4-6463-01A-11R-1723-07
	TCGA-DM-A1DB-01A-11R-A155-07
	TCGA-A6-2683-01A-01R-0821-07
	TCGA-NH-A6GB-01A-11R-A37K-07
	TCGA-AA-3818-01A-01R-0905-07
	TCGA-AA-A02F-01A-01R-A089-07
	TCGA-A6-5656-01A-21R-A278-07
	TCGA-AA-3680-01A-01R-0905-07
	TCGA-A6-5665-01B-03R-2302-07
	TCGA-A6-5665-01A-01R-1653-07
	TCGA-AA-A01S-01A-21R-A083-07
	TCGA-CM-5862-01A-01R-1653-07
	TCGA-AA-3555-01A-01R-0821-07
	TCGA-A6-5666-01A-01R-1653-07
	TCGA-G4-6317-01A-11R-1723-07
	TCGA-AA-3947-01A-01R-1022-07
	TCGA-A6-5661-01A-01R-1653-07
	TCGA-A6-5659-01A-01R-A278-07
	TCGA-AA-3846-01A-01R-1022-07
	TCGA-AA-3664-01A-01R-0905-07
	TCGA-AA-A02W-01A-01R-A00A-07
	TCGA-AA-3666-01A-02R-0905-07
	TCGA-D5-6922-01A-11R-1928-07
	TCGA-AY-6386-01A-21R-1723-07
	TCGA-CM-5864-01A-01R-1653-07
	TCGA-AA-3841-01A-01R-0905-07
	TCGA-A6-3809-01A-01R-A278-07
	TCGA-D5-6538-01A-11R-1723-07
	TCGA-CM-6677-01A-11R-1839-07
	TCGA-AA-3968-01A-01R-1022-07
	TCGA-DM-A282-01A-12R-A16W-07
	TCGA-AA-3864-01A-01R-1022-07
	TCGA-DM-A28C-01A-11R-A32Y-07
	TCGA-AZ-4682-01B-01R-A32Z-07
	TCGA-D5-6539-01A-11R-1723-07

Table S4. The gene list of candidate genes.

Gene symbol
HERPUD1
VAPA
SERPINH1
CYCS
SLMAP
PLOD3
SCD
VEGFA
CNPY3
ENTPD5
SRI
PPP1CB
IFI6
ZDHHC9
PSMA7
TRAP1
NBEAL2

Table S5. Hydrogen bond between CNPY3 and MDM2.

CNPY3 (red)	MDM2 (yellow)	Hydrogen bond length (A˚)
LYS-229	SER-22	3.4
LYS-229	GLU-25	2.8
LYS-244	SER-78	2.3
GLU-131	HIA-73	3.2
GLU-131	GLU-69	2.0
THR-132	ASP-68	3.2
ASN-135	GLN-71	2.4
TYR-149	ARG-65	2.6
TYR-149	GLY-87	3.4
LYS-124	ALA-43	3.1
SER-61	GLN-44	2.2
GLU-65	THR-47	2.7
HIS-272	THR-47	3.2
HIS-272	THR-47	2.7
GLN-265	SER-17	2.0
GLN-265	SER-78	2.9
GLN-265	ASP-84	2.3
ASP-261	LYS-98	3.3
GLY-263	SER-90	3.3
SER-257	THR-26	3.4
ASP-255	ARG-97	2.6
ASP-255	ARG-97	3.0
ASN-135	ASP-68	3.2
THR-132	GLU-69	2.0
THR-132	GLN-71	2.4
LYS-266	SER-78	2.9
LYS-266	ASP-84	2.3
SER-257	THR-26	3.4

ASP-255	SER-257	2.6
ASP-255	SER-257	3.0
ASP-109	SER-40	2.3
TYR-89	ARG-29	2.9
GLU-98	ARG-29	3.4
GLY-76	SER-78	3.0
TYR-77	SER-78	3.1
SER-241	ASN-79	2.0
SER-241	ASP-84	3.1

Supplementary Figure Legend

Figure S1. SREBP2 inhibits CRC in vivo

(A) Kaplan-Meier survival curve of overall survival analysis of colon cancer patients stratified by SREBP2 from Kaplan-Meier plotter. (B) The bar graph shows the percentage of CRC and neoplasia in the saline and Fatostatin-treated AOM/DSS induced CRC group. (C) Histograms show the levels of serum total cholesterol in the saline and Fatostatin-treated AOM/DSS induced CRC group.

Note: two-tailed $^{*}P < 0.05$ by an unpaired *t* test (B); data are presented as mean ± SD.

Figure S2. SREBP2 promotes the expression of CNPY3

(A) The Venn plot show CNPY3 is a candidate gene of SREBP2 from different databases. (B) Western blot showed pSREBP2 and nSREBP2 in HCT116 and RKO cells with Fatostatin or 25-OHC treatment, using β-tubulin as a control. (C) The mRNA expression of SREBP2 (up) and CNPY3 (down) in HCT116 (left) and RKO (right) cells after SREBP2 overexpression, using GAPDH as a control. (D) Western blot showed pSREBP2, nSREBP2 and CNPY3 in HCT116 and RKO cells overexpressed HA-SREBP2 plasmids, using β-tubulin as a control. (E) The mRNA expression of SREBP2 (up) and CNPY3 (down) in HCT116 (left) and RKO (right) cells after SREBP2 knockdown, using GAPDH as a control. (F) Western blot showed pSREBP2, nSREBP2 and CNPY3 in HCT116 and RKO cells with SREBP2 knockdown, using β-tubulin as a control.

Note: two-tailed $^{*}P < 0.05$ by an unpaired *t* test (B, D); 25-OHC, 25-Hydroxycholesterol; data are presented as mean ± SD.

Figure S3. SREBP2 is a transcriptional regulator of CNPY3

Mutated binding site of the transcription factor SREBP2 to the CNPY3 promoter and promoter activity of CNPY3 and mutant CNPY3.

Note: two-tailed $^{*}P < 0.05$ by an unpaired *t* test; data are presented as mean ± SD.

Figure S4. CNPY3 expression correlates with cholesterol levels

(A) Representative IHC of SREBP2 in CRC tissues from patients with low and high SREBP2 levels.

(B) Spearman's correlation analysis of total cholesterol and CNPY3 in CRC patients.

Figure S5 CNPY3 does not regulate cholesterol biosynthesis

(A-B) Western blot showed CNPY3 in HCT116 and RKO cells with CNPY3 overexpression (A) and knockdown (B), using β-tubulin as a control. (C-D) The mRNA expression of CNPY3 in HCT116 (left) and RKO (right) cells after CNPY3 overexpression (C) and knockdown (D), using GAPDH as a control. (E) Representative imagines (up) and semi-quantitative analysis (down) of lipid droplets in HCT116 and RKO cells after CNPY3 knockdown were determined by BODIPY staining. (F) Histograms showed the concentration of cholesterol within HCT116 (left) and RKO cells (right) after CNPY3 overexpression (up) or knockdown (down).

Note: two-tailed $*P < 0.05$ and ns by an unpaired t test (C, D, E, F); ns, not significant; data are presented as mean ± SD.

Figure S6. CNPY3 is upregulated in multiple cancers

(A) Boxplot of CNPY3 mRNA expression levels in multiple human cancers and normal tissues from the TCGA database in TIMER. (B-C) The mRNA (B) and protein (C) levels of CNPY3 in NCM460, HCT116, HT29, RKO, SW480, and DLD1 cells.

Note: CNPY3, Canopy FGF signaling regulator 3; ACC: Adrenocortical carcinoma; BLCA: Bladder Urothelial Carcinoma; BRCA: Breast invasive carcinoma; CHOL: Cervical and endocervical cancers; CESC: Cholangiocarcinoma; COADREAD: Colon adenocarcinoma and Rectum adenocarcinoma; COAD: Colon adenocarcinoma; DLBC: Lymphoid Neoplasm Diffuse Large B-cell Lymphoma; ESCA: Esophageal carcinoma; GBM: Glioblastoma multiforme; HNSC: Head and Neck squamous cell

carcinoma; KICH: Kidney Chromophobe; KIRC: Kidney renal clear cell carcinoma; KIRP: Kidney renal papillary cell carcinoma; LAML: Acute Myeloid Leukemia; LGG: Brain Lower Grade Glioma; HCC: Hepatocellular Carcinoma; LUAD: Lung adenocarcinoma; LUSC: Lung squamous cell carcinoma; MESO: Mesothelioma; OV: Ovarian serous cystadenocarcinoma; PAAD: Pancreatic adenocarcinoma; PCPG: Pheochromocytoma and Paraganglioma; PRAD: Prostate adenocarcinoma; READ: Rectum adenocarcinoma; SKCM: Skin Cutaneous Melanoma; STAD: Stomach adenocarcinoma; TGCT: Testicular Germ Cell Tumors; THCA: Thyroid carcinoma; UCEC: Uterine Corpus Endometrial Carcinoma; UCS: Uterine Carcinosarcoma; UVM: Uveal Melanoma; two-tailed $*P < 0.05$ by an unpaired t test (B); data are presented as mean ± SD.

Figure S7. CNPY3 promotes CRC cell invasion ability

(A-B) Representative imagines of wound healing assay of HCT116 (A) and RKO (B) cells treated with CNPY3 overexpression (left) and quantitative statistics (right).

Note: two-tailed $*P < 0.05$ by an unpaired t test (A, B); data are presented as mean ± SD.

Figure S8. Fatostatin inhibits the proliferative effects of CNPY3

(A-B) Representative images (left) and quantitative statistics (right) of colony formation assay of HCT116 (A) and RKO (B) cells with CNPY3 overexpression and controls after Fatostatin treatment.

Note: two-tailed $*P < 0.05$ by a Tukey's multiple comparisons test (A, B); data are presented as mean ± SD.

Figure S9. CNPY3 regulates the transcriptional level of MDM2

(A) Bubble chart shows the KEGG pathway analysis from TCGA based on the comparison of CNPY3 expression. (B) Western blot showed pSMDM2 in HCT116 and RKO cells with Terbinafine treatment, using β-tubulin as a control.

Figure S10. CNPY3 forms Condensate

Representative immunofluorescence images show the endogenous CNPY3 condensates of RKO and HCT116 cells.

Figure S11. CNPY3 regulates the p53 pathway to promote CRC

(A) GSEA analysis of p53 signaling pathway for KEGG enrichment using TCGA-COAD/ TCGA-READ cohorts divided by CNPY3 expression. (B) Bubble chart shows the KEGG pathway analysis based on the comparison of two subtypes of genes related to cholesterol synthesis.

Figure S12. CNPY3 binds to MDM2 to mediate degradation of p53.

(A) CNPY3-MDM2-P53 trimeric molecular docking pattern. (B) Endogenous Co-IP was performed in HCT116 cell lysates using an anti-CNPY3 antibody (up) and anti-p53 antibody (down), compared with control anti-IgG. (C) Representative confocal images of colocalization between CNPY3 and p53 and co-localization analysis in RKO cells.

Figure S13. CNPY3 inhibits p53 expression

(A-B) The expression level of p53 in the HCT116 and RKO cells with SREBP2 overexpression (A) and knockdown (B). (C) Representative IF imagines exhibited p53 (green) after CNPY3 overexpression in HCT116 and in RKO cells.

Figure S14. CNPY3 could inhibit apoptosis in CRC

(A-B) Apoptosis assays (left) and quantitative statistics (right) of HCT116 and RKO with CNPY3 overexpression (A) and CNPY3 knockdown (B).

Note: two-tailed $*P < 0.05$ by an unpaired *t* test (A, B); data are presented as mean ± SD.

Figure S15. Overexpression of CNPY3 inhibits the p53 signaling pathway in vivo

Quantitative statistics of IHC staining of CNPY3 (left), p53 (middle), and BAX (right) expression

in HCT116 tumor xenografts.

Note: two-tailed *$P < 0.05$ by an unpaired t test; data are presented as mean ± SD.

Figure S16. Verification of p53 knockout in HCT116 cells

(A) The p53 protein expression in HCT116 and p53-/- HCT116 cells, using β-tubulin as a control. (B) The protein levels of CNPY3 in p53-/- HCT116 cells with CNPY3 overexpression (left) and CNPY3 knockdown (right), using β-tubulin as a control.

Figure S17. CNPY3 loses regulation of CRC carcinogenic effects after p53 knockout

(A) Quantitative analysis of colony formation assay of p53-/- HCT116 cells treated with CNPY3 knockdown, CNPY3 overexpression, or Fatostatin treatment. (B) Semi-quantitative analysis of of EdU incorporation of p53-/- HCT116 cells treated with CNPY3 knockdown, CNPY3 overexpression, or Fatostatin treatment.

Note: ns by an unpaired t test (A, B); ns, not significant; data are presented as mean ± SD.

Figure S18. CNPY3 has a worse prognosis in patients with wild p53 rather than mutant p53

(A) Kaplan-Meier survival curve of overall survival analysis of all colon cancer patients stratified by CNPY3 from Kaplan-Meier plotter. (B-C) Kaplan-Meier survival curve of overall survival analysis of colon cancer patients with wild-type p53 (B) and mutant p53 (C) stratified by CNPY3 from Kaplan-Meier plotter.

Figure S19. The combination of Nutlin-3 and Fatostatin increases the inhibitory effect of CRC

(A) Representative images (left) and semi-quantitative analysis (right) of IHC staining of CNPY3 and p53 in AOM/DSS-induced CRC model with saline and Fatostatin treatment. (B) The protein levels of CNPY3 and p53 in AOM/DSS-induced CRC model with saline and Fatostatin treatment, using β-tubulin as a control. (C) CCK8 assays of HCT116 (left) and RKO cells (right) with DMSO,

Fatostatin, Nutlin-3 and combination of Fatostatin and Nutlin-3, respectively. (D) The protein levels of p53 and BAX in HCT116 (left) and RKO cells (right) with DMSO, Fatostatin, Nutlin-3 and combination of Fatostatin and Nutlin-3, respectively, using β-tubulin as a control. (E-F) Apoptosis assays (E) and colony assays (F) of HCT116 and RKO cells with DMSO, Fatostatin, Nutlin-3 and combination of Fatostatin and Nutlin-3, respectively.

Note: two-tailed *$P < 0.05$ by an unpaired *t* test (A), a Mix-effects analysis (C), and a Tukey's multiple comparisons test (E, F); data are presented as mean ± SD.
